# Health seeking behaviours and private sector delivery of care for non-communicable diseases in low- and middle-income countries: a systematic review

**DOI:** 10.1186/s12913-023-10464-0

**Published:** 2024-01-23

**Authors:** Callum Brindley, Nilmini Wijemunige, Charlotte Dieteren, Judith Bom, Bruno Meessen, Igna Bonfrer

**Affiliations:** 1 Erasmus School of Health Policy and Management, Erasmus University Rotterdam, P.O. Box 1738, 3000 DR Rotterdam, The Netherlands; 2 Erasmus Centre for Health Economics Rotterdam (EsCHER), Erasmus University Rotterdam, P.O. Box 1738, 3000 DR Rotterdam, The Netherlands; 3 Institute for Health Policy, Colombo, Sri Lanka; 4 The World Health Organization, Geneva, Switzerland

**Keywords:** Non-communicable disease, Private sector, Health seeking, Low-and-middle-income countries, Healthcare providers

## Abstract

**Background:**

Globally, non-communicable diseases (NCDs) are the leading cause of mortality and morbidity placing a huge burden on individuals, families and health systems, especially in low- and middle-income countries (LMICs). This rising disease burden calls for policy responses that engage the entire health care system. This study aims to synthesize evidence on how people with NCDs choose their healthcare providers in LMICs, and the outcomes of these choices, with a focus on private sector delivery.

**Methods:**

A systematic search for literature following PRISMA guidelines was conducted. We extracted and synthesised data on the determinants and outcomes of private health care utilisation for NCDs in LMICs. A quality and risk of bias assessment was performed using the Mixed Methods Appraisal Tool (MMAT).

**Results:**

We identified 115 studies for inclusion. Findings on determinants and outcomes were heterogenous, often based on a particular country context, disease, and provider. The most reported determinants of seeking private NCD care were patients having a higher socioeconomic status; greater availability of services, staff and medicines; convenience including proximity and opening hours; shorter waiting times and perceived quality. Transitioning between public and private facilities is common. Costs to patients were usually far higher in the private sector for both inpatient and outpatient settings. The quality of NCD care seems mixed depending on the disease, facility size and location, as well as the aspect of quality assessed.

**Conclusion:**

Given the limited, mixed and context specific evidence currently available, adapting health service delivery models to respond to NCDs remains a challenge in LMICs. More robust research on health seeking behaviours and outcomes, especially through large multi-country surveys, is needed to inform the effective design of mixed health care systems that effectively engage both public and private providers.

**Trial registration:**

PROSPERO registration number CRD42022340059.

**Supplementary Information:**

The online version contains supplementary material available at 10.1186/s12913-023-10464-0.

## Introduction

Globally, non-communicable diseases (NCDs) are the leading cause of mortality and morbidity placing a huge burden on individuals, their families and health systems [1, 2]. Four-fifths of NCDs occur in low- and middle-income countries (LMICs) and over two-fifths of deaths from NCDs in LMICs affect people younger than 70 years of age [3, 4]. This rising disease burden calls for policy responses that engage the entire health care system, including the non-negligible share of private providers. Evidence on private health care seeking behaviour for NCDs is scattered though, and the appropriate role of the private sector in the delivery of health care has been heavily debated [5–9]. Proponents argue that the private sector is often more efficient than the public sector, [10, 11] can offer better quality of care, [11, 12] and adds capacity to the health system thereby increasing access [13, 14]. Critics counter that the profit motive leads to an oversupply of higher cost services of variable quality rather than what is needed and cost-effective [15–17]. Additionally, higher quality private health services are more accessible to advantaged groups thereby contributing to inequalities [18]. Putting aside this polemic, a pragmatic approach for governments is to regulate the health sector as a whole and encourage a mix of public and private providers that ensures accessible, good quality, affordable care [19–22].

This study aims to synthesize the scattered evidence on how people with NCDs choose their healthcare providers in LMICs, and the outcomes of these choices, with a focus on the understudied private sector. In particular, we concentrate on the contextual and individual factors that influence provider choice, patterns of utilisation, quality of care and financial protection. Existing literature on the private sector in LMICs has largely focused on infectious diseases, and maternal and child health rather than chronic conditions. Data on reproductive, maternal, newborn and child health (RMNCH) from nationally representative Demographic and Health Surveys (DHS) since 1990 for over 50 LMICs suggests that the private sector is an important but not dominant provider, with private health care utilisation varying across countries, provider type, level of care and by disease/condition [23, 24]. Health seeking behaviours for RMNCH, however, are potentially different to those for other diseases. For example, RMNCH services has been heavily supported by the international community for several decades and prioritised by national health authorities (including the removal of user fee) [25, 26]. Earlier systematic reviews of the comparative performance of public versus private providers in LMICs have not found differences in efficiency or health outcomes across the private and public health care sectors [16, 27]. These reviews do find that the private sector performs better in aspects of care delivery such as responsiveness and hospitality towards patients [12, 28]. A systematic review of financial protection in LMICs looked at chronic illnesses separately from other illnesses, but did not consider variation by public versus private provider. An understanding of the determinants and outcomes of NCD care for both public and private providers is therefore timely and valuable [29, 30].

Strategies for the prevention and control of NCDs call for population wide interventions to promote healthy behaviours and reduce risk factor exposure, combined with efficient, integrated health services to screen, detect and provide long-term treatment [30]. This implies coordination among providers to ensure a continuity of care, and a people-centered approach that empowers individuals to self-manage their chronic condition [31]. In many LMICs, providing this model of care remains a challenge because health services have been traditionally oriented towards episodic, acute care particularly for infectious diseases and RMNCH [32–36]. Often, national NCD strategies also focus on the public sector, excluding the private sector, despite the two being entwined elements of the health sector [37–39]. Benefit packages in many LMICs only offer limited coverage for NCDs, such that patients must pay out-of-pocket even in public health facilities. This risks discouraging or delaying health seeking, leading to ineffective care, poor health outcomes and catastrophic health spending [40, 41]. Better understanding health seeking behaviours for NCDs, including private care use, can inform the the effective design and implementation of interventions across the different building blocks of the health system [42]. This review will thus inform the organisation of mixed health systems in the pursuit of Universal Health Coverage (UHC) and the achievement of Sustainable Development Goal (SDG) target 3.4 – a one-third reduction in premature mortality from NCDs by 2030.

## Methods

### Research question

This systematic review on the determinants and outcomes of private health care utilisation is built around the questions – *What factors influence private health care seeking behaviour for individuals with NCDs in LMICs? From whom is health care obtained, what are the patterns of utilization, what are the determinants that influence the use of private as opposed to public health care, and what are the outcomes of this health care seeking?* This systematic review allows us to determine the size and nature of the current literature and to identify major knowledge gaps that relate to mixed health care systems with entwined public and private providers of NCD care in LMICs [43]. The protocol has been published [44] and was registered on 15 June 2022 with PROSPERO (CRD42022340059). Its reporting is guided by the Preferred Reporting Items for Systematic Reviews and Meta-Analyses and its extension for literature searches PRISMA-S [45, 46].

### Eligibility criteria

Table 1 outlines the Populations, Interventions, Control, Outcomes, Timeframe, Setting (PICOTS) criteria applied. We focused on adults and defined the scope of health care to encompass the prevention, diagnosis, treatment and/or management of disease. We restricted our search to the four largest groups of NCDs responsible for over 80% of all premature NCD deaths, i.e. cardiovascular diseases, diabetes, cancers, and chronic respiratory diseases. With regard to cancers, we limited our scope to the top five cancers with the greatest disease burden for each sex [3]. Following the World Health Organization’s (WHO) operational definition we defined the private health sector as individuals and organizations that are neither owned nor directly controlled by governments, and are involved in the provision of health services (i.e. informal and formal providers as well as for-profit and non-profit entities, which may include privately owned providers that receive public funding) [48]. Our outcomes of interest were the determinants and outcomes of health seeking behaviour. We focused on the timeframe from January 2010 to June 2022, which is defined by large increases in the NCD disease burden in LMICs and the first High-Level Meeting of the UN General Assembly on the Prevention and Control of Non-communicable Diseases [3, 49]. This review adds to a broader systematic review into the private provision of health services in LMICs conducted in 2011 [50]. Lastly, the study concentrated on settings in LMICs as defined by the World Bank classification for 2022 (i.e. countries with a gross national income per capita of $4095 or less) [47].

**Table 1:** PICOTS criteria

Population	Adults and households at risk, or diagnosed with at least one of the following non-communicable diseases: cardiovascular diseases, diabetes, chronic respiratory diseases or cancers (tracheal, bronchus and lung; colon and rectum; pancreas; stomach; breast and prostate)
Interventions	Private sector provision of health care services that involves the prevention, diagnosis, treatment and/or management of non-communicable disease
Comparator	Studies focused on private sector exclusively, or both private and public sectors
Outcomes	Determinants and outcomes of health care seeking behaviour, at the individual and population level
Timeframe	1 January 2010 to 30 June 2022
Setting	Low-income and middle-income countries following the World Bank 2022 classification [47]

For inclusion, studies had to adhere to all elements defined in Table 1 and had to be published in English in a peer-reviewed journal. We considered both qualitative and quantitative empirical studies. Editorials, commentaries, reviews, and protocols were excluded.

Studies had to address private sector health care and could do so in a comparison with the public sector. We excluded studies that only reported aggregated data (i.e. private and public health sector combined) and studies that focused on outcomes in the private sector without a comparison to the public sector. Lastly, we excluded vaccination programmes associated with NCDs (e.g., human papillomavirus vaccine) since these are commonly government-led, as well as settings with irregular contextual circumstances (e.g., conflict, economic or political crises) because these are not generalizable.

### Search strategy

We conducted the search using Embase, Medline, Web of Science Core Collection, EconLit, Global Index Medicus and Google Scholar (see Supplementary Material (SM) Text S.1 for an extended description of the strategy and selection process). For all criteria we defined key terms and relevant thesaurus terms tailored to the specific database.

### Selection process

The articles resulting from the search were screened by a team of two pairs (CB-JB & NW-CD) using Rayyan Reference Manager. Each article was title-abstract screened independently by both team members using the eligibility criteria. Studies adhering to all eligibility criteria were full-text reviewed and reasons for exclusion at this stage were documented. For both stages, differences in screening results were discussed and resolved by dialogue.

### Quality assessment

We used the Mixed Methods Appraisal Tool (MMAT) to assess the methodological quality and risk of bias of the included full-text articles [51]. Each full-text article was assessed by a team of two pairs (CB-JB & NW-CD) with one person taking the lead and the second person reviewing for completeness and accuracy. Discrepancies and disagreements were resolved through discussion.

### Data extraction and synthesis

We piloted and refined a data extraction framework including *study details* such as authors, title and year; and *study characteristics* such as research design, sample size, studied country, disease and provider type. We also extracted the *determinants of selecting a health care provider*, which we grouped into three categories: 1) *individual factors* such as demographics and health; 2) *contextual factors* such availability and accessibility of health care; and 3) *perception of providers* such as quality and competence. Lastly, we extracted *outcomes of selecting a health care provider*, which we distinguished into three categories: 1) *patterns of utilization* including characteristics of patients and equity issues; 2) *quality of care* including the Donabedian model [52] domains of process and outcomes as well as patient satisfaction and empowerment; and 3) *spending and financial protection* as captured by indicators such as catastrophic health spending and impoverishment. Two individuals read the full-text of included articles, with one person taking the lead to extract the data, and the second person reviewing this work. We synthesised the results into the aforementioned three categories of determinants of selecting a health care provider (individual factors, contextual factors and perception of providers) and three categories of outcomes of selecting a health care provider (patterns of utilization, quality of care, and spending and financial protection). We also analysed the interaction of health seeking determinants and outcomes and prepared descriptive statistics on the included studies and their outcomes. The team met to discuss and analyse the data extracted, which we consolidated using a descriptive synthesis including a summary of the evidence, gaps, and limitations. We did not perform a meta-analysis because the study characteristics such as design type, setting, intervention and outcome, were too hetergeneous.

## Results

### Study characteristics

**Fig. 1:**
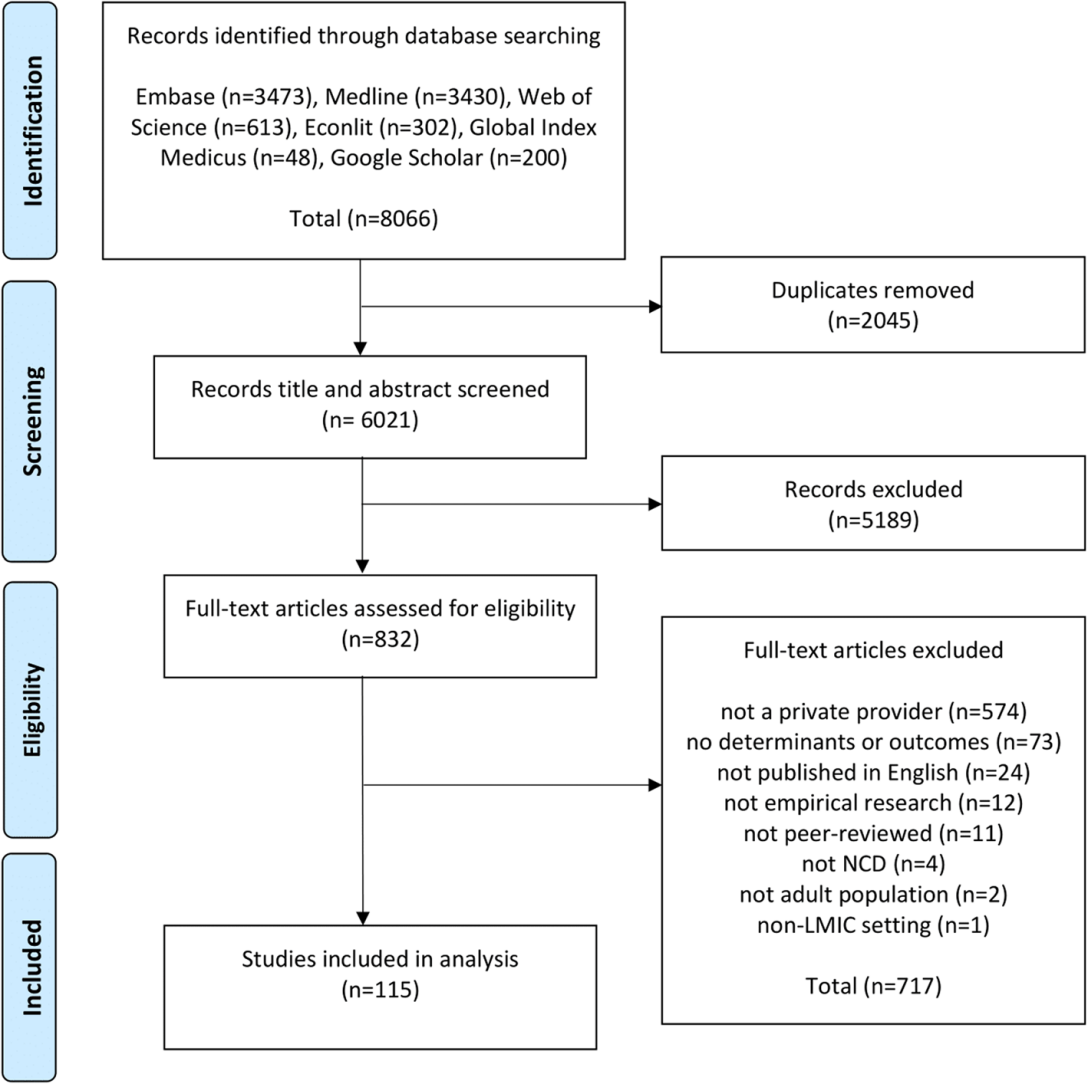
Flow diagram of study selection

Figure 1 shows the PRISMA flow diagram. In total, we selected 115 studies for inclusion (see SM Table S.1). Of these included studies, more than 70% were published in the last 5 years (i.e., 2017–2022).

**Fig. 2:**
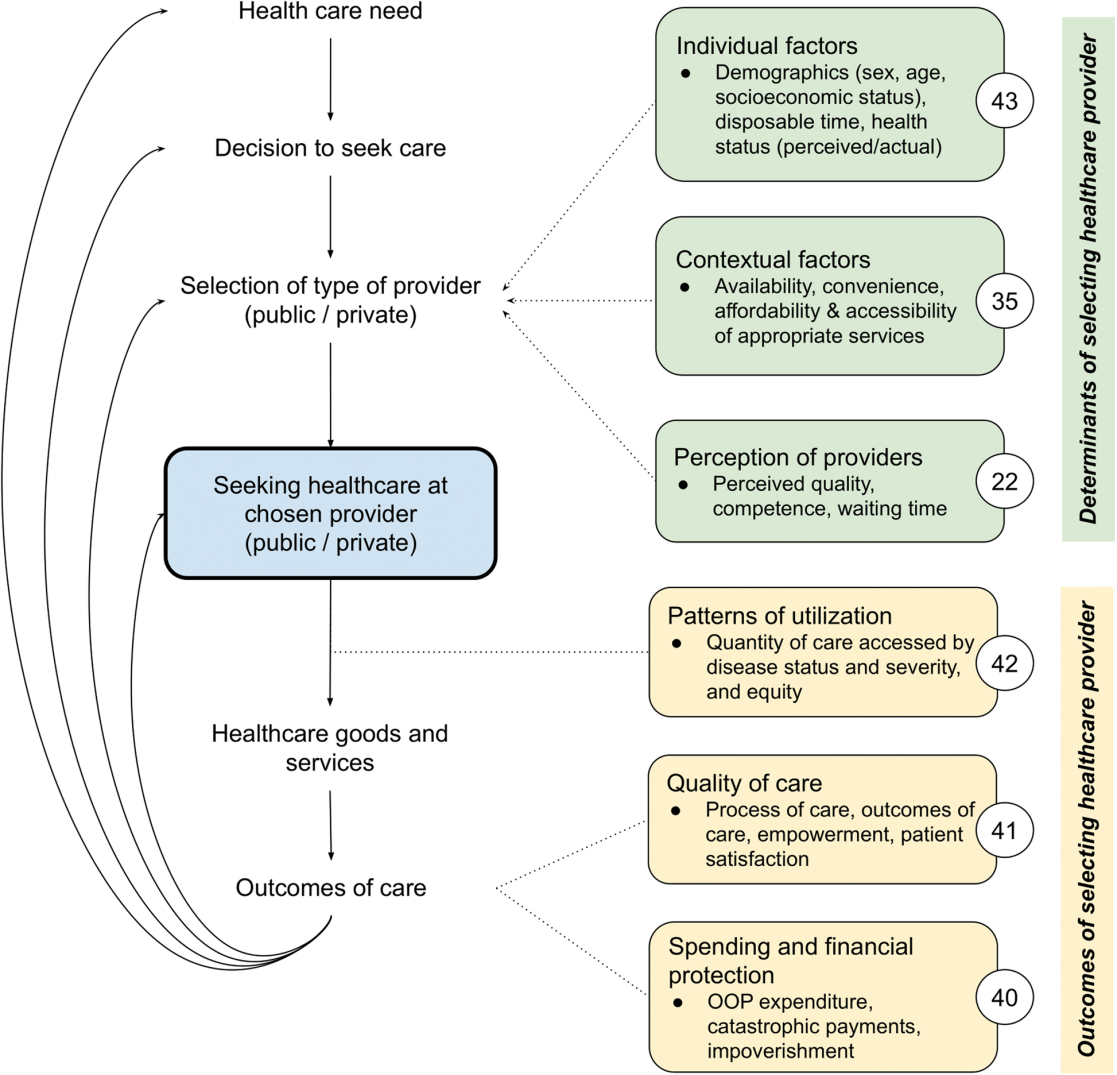
Determinants and outcomes along the patient journey of seeking private health care for NCDs. Note: Numbered circles in Fig. 2 indicate how many papers discussed the determinant or outcome

Figure 2 depicts our conceptual framework of the determinants and outcomes along the patient journey of seeking private health care for NCDs. Of the 115 included texts, 67 studies discussed determinants: individual factors (*n* = 43),contextual factors (*n* = 35) and perception of providers (*n* = 22). There were 87 studies that discussed outcomes: patterns of utilization (*n* = 42), quality of care (*n* = 41), and/or spending and financial protection (*n* = 40).

**Fig. 3:**
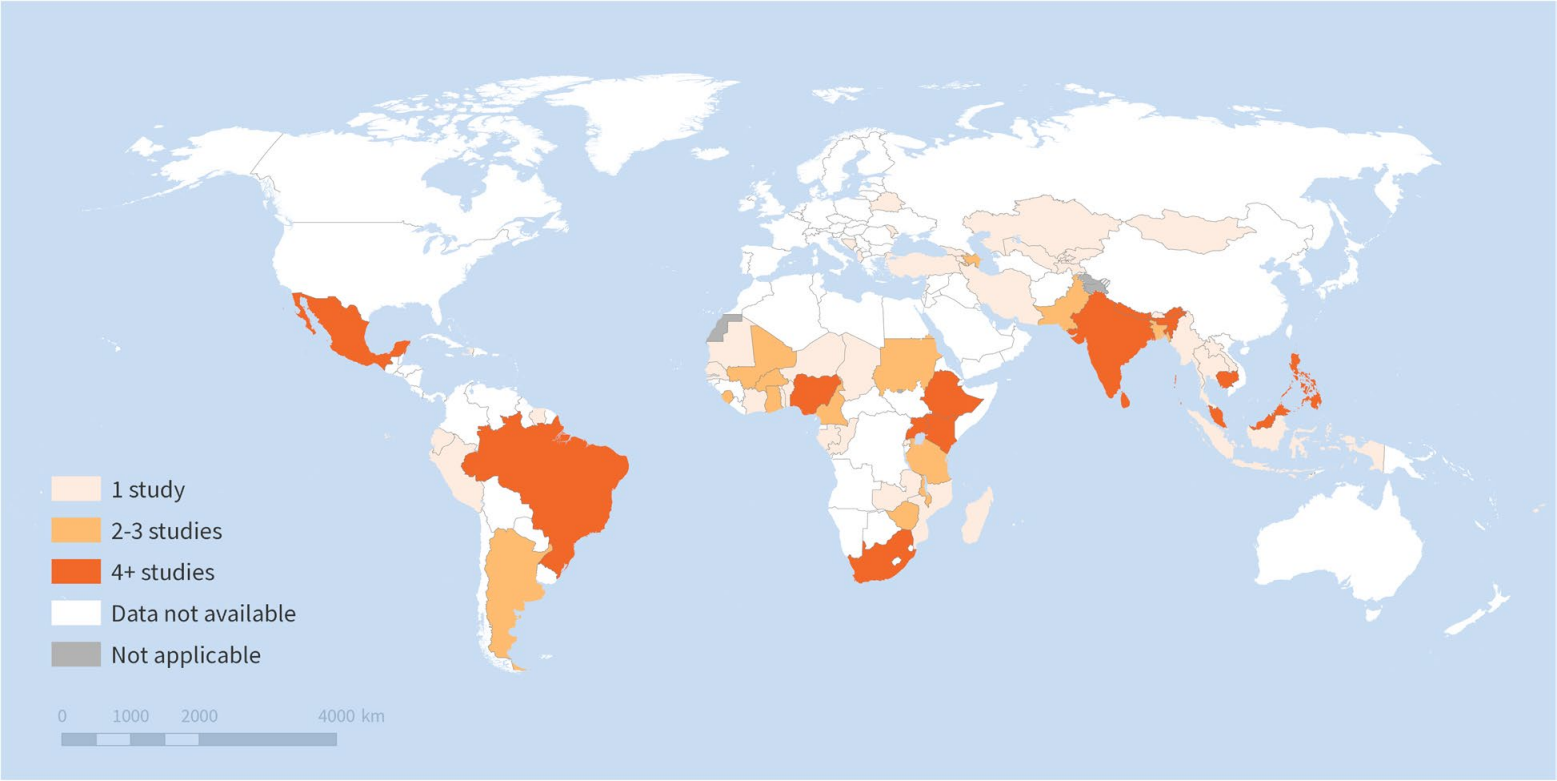
Low- and middle-income countries covered by selected studies

Figure 3 shows the LMICs included in the selected studies. In total, the studies covered 66 LMICs of which only 13 were low-income. The breakdown of countries by World Bank income group did not notably change if the income classification of the study year was used instead of the 2022 income classification. Almost all of the studies focused on a single country setting: only 4 of the 115 were multi-country studies [53–56]. Moreover, 64 of 111 studies with a single country setting focused on just 6 countries, India (*n* = 27), Brazil (*n* = 16), Kenya and South Africa (*n* = 6 each), Sri Lanka (*n* = 5) and Nepal (*n* = 4). There were no included studies on China because these lacked terminology to determine provider ownership.

**Fig. 4:**
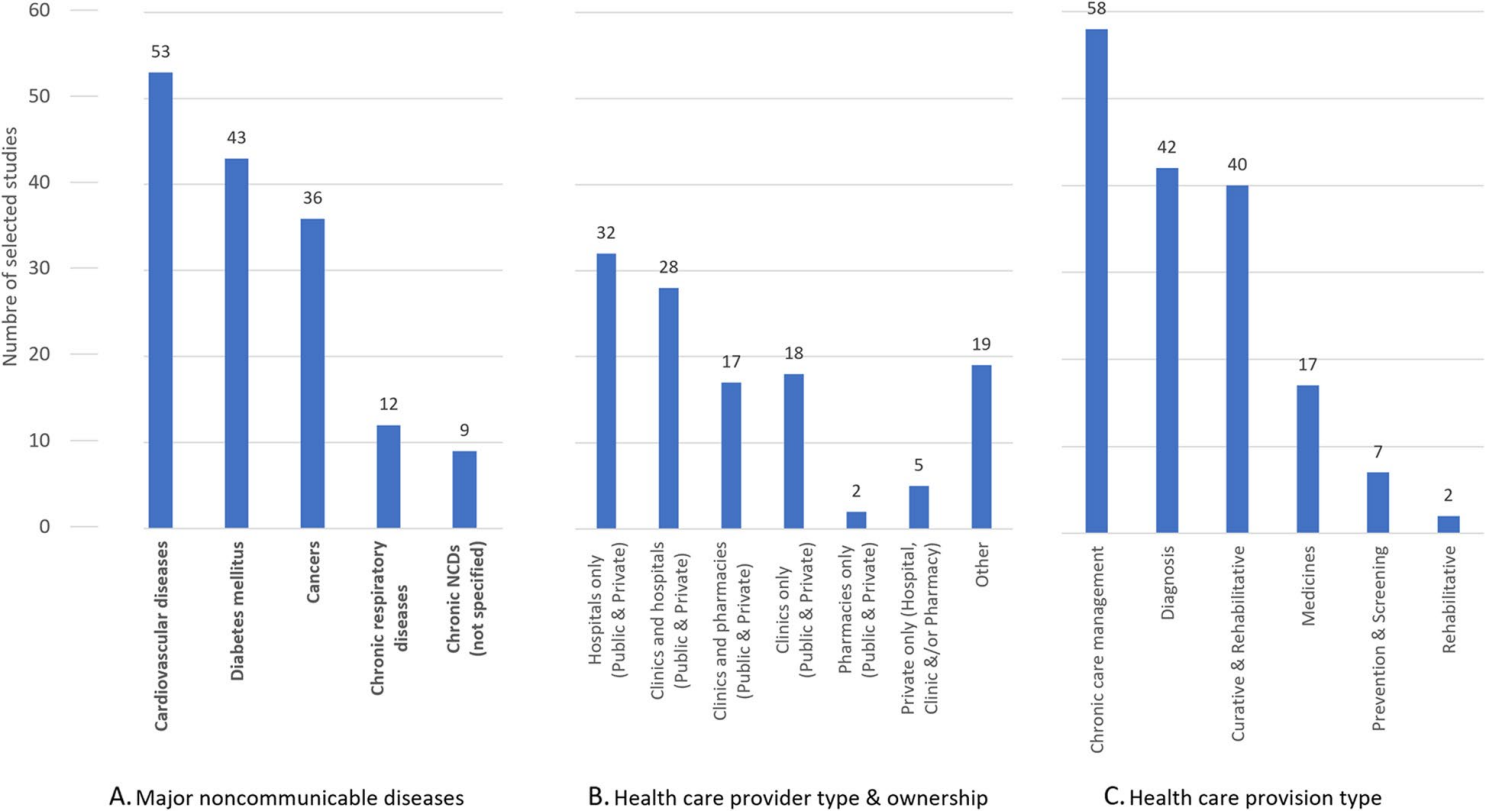
Major noncommunicable diseases, provider type and health care covered by selected studies. Note: Individual studies may address multiple countries, disease, providers, and types of health care provision. Diseases were restricted by our eligibility criteria to four major groups of NCDs: cardiovascular diseases, cancers, respiratory diseases and diabetes. Cancers were further restricted to the top five cancers with the greatest disease burden for each sex. The category other in panel B consists of combinations of public and private provider types not otherwise listed

Figure 4 shows 88 studies focused on a single disease and 27 on multiple. Cardiovascular disease, diabetes, cancers, and chronic respiratory disease were the most studied. Over a third of the selected articles looked at a combination of provider types, another quarter focused exclusively on hospitals (*n* = 32). Almost all studies compared public and private sector providers (*n* = 110). We observe a close association between disease, provider type and care. For example, a majority of the studies looking at diabetes focused on the primary care providers of health clinics and/or pharmacies (*n* = 23), with an emphasis on diagnosis, chronic care management and medicines. Only 7 studies focused on prevention and screening.

### Assessment of quality

Over two-thirds of the studies applied a quantitative methodology including 62 cross-sectional, 17 cohort, 2 quasi-experimental designs, [57, 58] and 1 randomised controlled trial [59]. Nearly a third of studies had a qualitative (*n* = 19) or mixed-methods (*n* = 14) design. Surveys were the most common data source (*n* = 49), but only 18 of these studies used nationally representative samples. Often sample sizes were less than 1500 patients, purposively selected from specific providers or a subnational national region. Virtually all the qualitative and mixed-methods studies had sample sizes of less than 500 participants and relied heavily on interviews. While the MMAT appraisal questions don’t allow a comparison by research design, the average ratings were similar across the design types. The MMAT scores were considered during data synthesis and we gave greater emphasis to studies with larger, representative samples (see SM Table S.2 for an overview of the research designs and MMAT ratings).

### Determinants of health care decision

#### Individual factors

We categorised the determinants of health care decisions into three groups (Fig. 2). Regarding *individual factors*, we did not find an obvious set of characteristics that uniquely define private care users: individuals of all backgrounds visit private providers at different points along the care pathway. Many papers observe that individuals seeking care in the private sector often have a higher socio-economic status [60–72]. Patients presenting to public facilities have more multimorbidities, [73] belong to higher risk groups, [61, 74] and seek care at a more advanced stage of disease [74–76] (i.e. public providers tend to have a more complex case-mix). The ability to continue one’s care journey in the private sector also appears to be influenced by individual characteristics. For example, Risso-Gill et al. [77] found that individuals with mid-level incomes in Malaysia might use private care for acute illness but would move to the public sector when they develop a chronic condition. Indeed, transitioning between public and private facilities seems to occur often [77–81].

#### Contextual factors

We found that that *contextual factors* can play an important role in health care decisions. Many papers indicated that the availability of facilities, staff and health services were reasons to seek care in the private sector [82–87]. For example, multiple studies reported that frequent medicine shortages in public facilities drive individuals towards the private sector, [55, 72, 82, 88–96] and that even public providers themselves sometimes refer patients to private pharmacies [88, 91]. Additionally, accessibility and convenience are important determinants for seeking private care. For example, individuals tend to visit private providers because they are geographically closer [82, 97, 98] and have longer opening times [85, 96, 99]. Similarly, Elias et al. [88] found that patients in India prefer private facilities because they offer multiple services in one place, hence limiting the need to coordinate separate visits for consultation and diagnostics. Although poorer patients tend to rely heavily on public health facilities, Tripathy et al. [98] and Kujawski et al. [82] found that almost half of the poorest households in India sought care at private facilities for quality reasons despite considerably higher costs. While many of the included papers rely on small or non-representative datasets, their findings on the importance of service availability and convenience are consistent with those using larger, more representative surveys such as Tripathy et al. [98] and Kujawski et al. [82] Finally, ability to afford care was another determinant for treatment in the private sector. Many studies highlighted that prices are a reason for halting or limiting treatment, or a reason for staying in or moving to public facilities [78, 92, 93, 95, 96, 100]. A quasi-experimental study in Brazil [58] supported these findings by showing that increased (decreased) cost-sharing of medication reduced (increased) dispensing in private pharmacies. A randomised control trial (RCT) in Kenya [59] also found that reducing prices increased the uptake of NCD medicines in the public sector. However, this did not lead to an increase in medicine availability at the household level, most likely because of medicine shortages.

#### Perception of providers

Finally, *perception of providers* including the perceived quality of care was an important determinant for deciding to seek private health care. Many papers emphasized a higher perceived quality of care or trust in private facilities [77, 82, 88, 95–98, 101]. For example, Thomson et al. [95] mention that public hospitals in Sudan were often described as not being clean enough and chaotic compared to private facilities. Additionally, papers concerning Uganda [96] and Cambodia [101] respectively mention the quality of medicines and trust in the provider as reasons to purchase medication at private pharmacies. Moreover, many papers mention the long waiting times at public facilities as reason for seeking private care [82, 84, 85, 90, 95, 97, 98, 102]. Such quality concerns can be strong drivers for care seeking behavior. For example, Perera et al. [79] describes how individuals in Sri Lanka are willing to incur additional costs and travel further to obtain care from providers with higher perceived quality. Again, while most of these findings are based on qualitative interviews or small and non-representative datasets, they align with findings based on larger representative samples. Not all papers, however, conclude that private care is unanimously preferred. Qualitative work in the Philippines [103] and Uganda [80] suggest that individuals distrust private providers and believe their main aim is to make a profit.

### Outcomes of health care decisions

#### Utilization

Turning to the outcomes of health care decisions (see Fig. 2), we found that *utilization patterns* for people with NCDs varied greatly by disease and country, with little commonality across study settings. Several studies found that the public sector diagnoses more NCD patients [60, 104] and is more frequented for outpatient visits, [54, 62, 104] pharmacy services, [54] and inpatient care [105]. Other studies, however, showed higher use of the private sector for initial presentation with symptoms [97, 106], purchasing medications, [59, 60, 72] diagnostic services, [107] and disease management [101, 103, 108–110] even in settings with free health care at public facilities. In India, [65] one study reported that this pattern of predominant private use extended even to people with NCDs living in slums. Several studies found that many individuals transition between public and private providers, which affects their continuity of care [77–81]. Private facility use was reported to be higher in the better off, and public facility use higher in poorer patients [98, 111, 112]. For example, a large representative study by Jeyashree et al. [112] found that private NCD hospitalizations in India showed a pro-rich pattern. However, this study also showed pro-rich utilization of public facilities in several states. This pattern was more pronounced in rural areas but the study did not control for possible differences in prevalence rates across socioeconomic groups.

#### Quality of care and patient satisfaction

Considering *quality of care* including process, outcomes, patient satisfaction and empowerment, [52] we found that the included studies were heterogeneous, often small, and conclusions differed depending on disease, facility type, and which aspect of quality was assessed. A common theme was the private sector performing better on *process quality* for resource-intensive interventions such as those requiring more health worker time, specialised expertise, costly medications, or diagnostics. Such examples included thrombolysis for stroke patients, [70] interventions for acute coronary syndrome (ACS), post-ACS management, [76, 113, 114] and cancer treatment, [69, 107, 115, 116] and routine screening for diabetes complications [117]. Two studies also reported that patients in the private sector had access to new or expensive medications for cancer treatment [118, 119]. When it came to less complex and less resource intensive interventions, a number of studies [68, 69, 111] found that public sector facilities were able to perform as well or better than the private sector.

With regard to *outcomes of care*, a number of studies [70, 76, 113, 120–122] found that the private sector performed better than the public sector, but there were relatively few studies that controlled for disease stage, severity, risk factors, comorbidities or the background of patients, which are often different between the two sectors [61, 73–76]. One study in Malaysia that did control for such differences found that lower cancer survival rates in the public sector were unlikely to be related to disparities in treatment, highlighting the need for earlier diagnosis of the uninsured and people from lower socioeconomic classes [119]. There were virtually no studies on the impact of attending the public and private sector on use of preventative programs, however one multi-country study found that people who last visited the private sector had reduced odds of having a pap smear, but increased odds of having a mammogram [53].

Lastly, several studies reported that patients were more satisfied with the private sector for reasons related to time spent with the doctor, interpersonal quality, psychosocial support and cleanliness [77, 94, 111, 123, 124]. There were, however, other studies that suggested higher patient satisfaction with the care given in the public sector [64] especially the technical quality of public sector doctors [111]. In terms of empowerment, patients attending the private sector received better diabetes education, [120] had higher odds of better knowledge, attitudes and practices on hypertension [125].

#### Spending and financial protection

In most settings, private costs to patients were (far) higher in the private sector across a range of NCDs in inpatient and outpatient settings [65, 83, 98–100, 105, 109, 112, 126–132]. Many studies reported higher incidence of financial hardship in the private sector [83, 98, 133, 134], catastrophic expenditure [83, 98, 105, 134–136], and high burden relative to wages. Furthermore, two high quality studies found that costs in the private sector have been increasing more over time, relative to costs in the public sector [134, 137]. Although public sector costs were usually less, with free or lower doctor fees and bed charges, [98, 135] indirect costs such as transport, food, lodging, and childcare, contributed to a larger proportion of out-of-pocket expenditures for patients in the public sector in some studies, [84, 105, 131, 136] sometimes making the overall cost of accessing public healthcare more expensive [84, 105]. Similarly, compared to their private counterparts, public patients were sometimes found to spend more on medicines and diagnostic tests [105]; or faced a higher contribution to total expenditures from medicines, though total expenditure was still lower [83, 98]; or were compelled to spend large amounts on medications from the private sector due to unavailability in the public sector, [138] with authors stressing the importance of increasing availability of services, and generic and subsidized medicines to increase access and affordability of healthcare [83, 98, 105, 132, 138].

## Discussion

### Principal findings

There is a non-negligible share of NCD patients who seek care from private health care providers in LMICs.

However, there is currently limited evidence on patients’ pathways and the effectiveness, efficiency and cost of NCD care in these settings. We conducted a systematic review of the determinants and outcomes of private health care utilisation for NCDs in LMICs. Specifically, we aimed to detail the determinants that influence the choice of health care provider, and the outcomes of these choices. Our search resulted in 6021 articles of which 115 were included for analysis based on our eligibility criteria. We found that most of these studies were based on specific settings with findings pertinent to a particular context, disease and provider with few studies using a multi-country design or nationally representative samples. Moreover, most studies did not control for differences in provider and patient characteristics, which limits the interpretation of the findings to an association rather than a causal explanation of outcomes. Notwithstanding these important limitations in the designs of the included studies, we observed several trends in the determinants and outcomes of private health seeking for NCDs in LMICs.

Regarding the determinants that influence choice of health care provider, we found that individuals who seek care in the private sector are highly diverse. Nonetheless, regular utilisation of private facilities appears higher among the socioeconomically advantaged. Less advantaged individuals also seek private healthcare, especially from pharmacies to obtain medication. Patients arriving at private facilities tend to seek care at an earlier stage of disease; public patients tend to face more multimorbidities. The most common determinants for people to seek care for NCDs in the private sector are physical accessibility of facilities; availability of care (particularly services, staff and medication) and convenience. Households appear to seek care strategically using their knowledge of cost-sharing policies and services offered by different providers to navigate the system for different types of care. In many settings, individuals believe that private facilities provide better quality care and are willing to incur additional costs and travel farther to obtain this care. This perception of better quality was not universal though.

Regarding outcomes of private health care seeking, utilisation patterns for NCD care in the private and public sectors vary greatly by disease and country, and few generalisations can be made. Nevertheless, as NCD care pathways are typically fragmented, transitioning between public and private facilities is common. Moreover, shortages of medication in the public sector combined with high prices in the private sector often contributed to the intermittent treatment of chronic conditions. Many studies report that process quality appears to be higher in private facilities for clinical care typically requiring more resources. For clinical care that is less complex or less resource intensive, public-sector facilities may perform as well or better than the private sector. Interpersonal quality of care and time spent with the doctor appears to be higher in the private sector. However, most studies did not control for differences in provider and patient characteristics when comparing public and private care. Critically, this limits the interpretation of the findings to an association rather than a causal explanation. Finally, regarding spending and financial protection, we found that private costs to patients and incidence of financial hardship are far higher in the private sector across all of the major NCDs.

### Policy implications

These principal findings point to four policy implications. Firstly, quality can be improved in both public and private providers. Weaknesses in the service delivery of NCD care at certain levels of the public system have probably been key drivers in the use of private providers in many LMICs [139–141]. Evidence suggests that the public sector needs to be more accessible, reliable and convenient while the private sector needs to be more affordable. Governments must ensure that all providers in an entwined health system contribute to the objective of universal health coverage. Second, continuity of care can be improved through regulating the performance and outcomes of the health sector as a whole. This is crucial given the need for long-term management of NCDs and the reality of mixed health systems in which patients transition between providers. Examples of possible initiatives might include the introduction of a personal health record system or provider payment mechanisms incentivising coordination and continuity of care across different providers. Third, supply-side reforms to provide a package of essential NCD interventions should be complemented by demand-side reforms that ensure health care is also affordable. For example, extending coverage for NCDs in benefits packages would help lower the risk of financial hardship and catastrophic spending noted in this review. Lastly, policy makers should prioritise the availability of affordable essential medicines for NCDs (e.g. strategic procurement, dispensing in public facilities, subsidised prices, reimbursement). Evidence from LMICs suggest that supply shortages and high costs of medicines especially when paid out-of-pocket are key problems in the effective management of chronic conditions.

### Evidence gaps

This systematic review found that research on health care seeking for NCDs in the private sector is underdeveloped. We identified five major evidence gaps. Firstly, most of the existing literature is based on specific settings with findings pertinent to a particular country context, disease and provider, which limits generalisability. Among the studies that directly compared private and public providers, relatively few controlled for differences in provider and patient characteristics. This gap points to the need for more research with an experimental design as well as large cross-country studies with representative national samples.

The second evidence gap is the focus on a relatively small group of LMICs, mostly in Africa and South-East Asia, with sparce research from the regions of Central Asia and the Middle East. The existing literature covered close to half of low-income countries, but the majority of studies still focus on middle-income settings. Chronic respiratory diseases also appear under researched, and there appears to be limited literature on patient empowerment, which is central to long-term management of NCDs. One could hypothesize that the different incentives faced by public and private-for-profit providers might lead to different commitments to educating patients on self-managing chronic conditions. Future research into health seeking might also do well to distinguish between people with and without a diagnosis.

Third, there is a lack of research into the informal private sector. The included studies focused almost exclusively on the formal, for-profit private sector despite our search strategy using a broader definition including non-profits, non-government organisations, charities, faith-based providers and traditional healers. This study area appears to be under researched, but a priority given that the informal sector tends to be monitored and regulated even less than the formal private sector. A related unresearched area of study would be to investigate the effects of channelling public funds to private sector providers of NCD care – none of the included studies specifically looked at contracting out by the public system.

The fourth major evidence gap is the limited evidence on individuals’ out-of-pocket spending on NCD care in private facilities. Although many studies reported that private costs to patients were far higher in the private sector than the public sector, we did not find any studies that evaluated out-of-pocket spending on NCDs incurred in the private sector as opposed to elsewhere. This is consistent with the scoping review of Rahman et al. [142] that there were no financial protection studies on chronic illnesses for low-income countries. The inclusion of expenditure by disease groups in national health accounts [143] and studies such as the iHOPE project [144] will contribute to addressing this gap but more research is still needed especially on the accumulation of bills for households with chronic illnesses.

Fifth, there is a need for impact evaluations of reforms to promote continuity of care and coordination between private and public providers. This review found that NCD care pathways are typically fragmented and that transitioning between public and private facilities is common. Frameworks for the continuity and coordination of care exist, usually with a focus on primary care [30, 31, 145, 146]. McPake and Hanson have also outlined approaches to managing the public-private mix of providers to achieve universal health coverage [19]. Impact evaluations of health system reforms with these aims (e.g., accreditation, integrated information systems, provider payment mechanisms) would strengthen the evidence base and help policy makers in LMICs.

### Limitations

Although we consider all LMICs, this systematic review was limited to studies published in English. We also limited our attention to literature published since 2010 but adopted a broad definition of the private sector to capture its breadth and complexity. A priori, we have not been able to examine closely the health seeking behaviours of individuals in LMICs who are undiagnosed or have foregone care for NCDs. This remains an important but understudied group. Lastly, our use of the MMAT to assess quality meant that we could include quantitative, qualitative and mixed-methods research designs but they are not easily compared. We were unable to perform a meta-analysis because of the highly diverse study characteristics, including design type, setting, intervention and outcome.

## Conclusions

The burden of NCDs is rising fast in LMICs. People living with chronic conditions have agency and seek care across the spectrum of providers. Health policy solutions for NCDs are more complex than what has worked for communicable diseases and for reproductive, maternal, newborn and child health. This calls for more research, and certainly interdisciplinary collaboration. We need quantitative descriptive studies to measure the scale of the numerous issues, qualitative studies to understand causal pathways and interventional studies to test and validate possible solutions which might work across the spectrum of providers.

### Supplementary Information

**Additional file 1.**

## Data Availability

The datasets used and/or analysed during the current study available from the corresponding author on reasonable request.

## Abbreviations

ACS: Acute coronary syndrome
DHS: Demographic and Health Surveys
LMIC: Low- and middle-income country
MMAT: Mixed Methods Appraisal Tool
NCD: Non-communicable disease
RMNCH: Reproductive, maternal, newborn and child health
PICOTS: Populations, Interventions, Control, Outcomes, Timeframe, Setting
PRISMA: Preferred Reporting Items for Systematic Reviews and Meta-Analyses
RCT: Randomised control trial
SDG: Sustainable Development Goal
UHC: Universal Health Coverage
WHO: World Health Organization
