# Correction: Health seeking behaviours and private sector delivery of care for non-communicable diseases in low- and middle-income countries: a systematic review

**DOI:** 10.1186/s12913-024-10713-w

**Published:** 2024-02-22

**Authors:** Callum Brindley, Nilmini Wijemunige, Charlotte Dieteren, Judith Bom, Bruno Meessen, Igna Bonfrer

**Affiliations:** 1 Erasmus School of Health Policy and Management, Erasmus University Rotterdam, 3000 DR Rotterdam, P.O. Box 1738, The Netherlands; 2 Erasmus Centre for Health Economics Rotterdam (EsCHER), Erasmus University Rotterdam, 3000 DR Rotterdam, P.O. Box 1738, The Netherlands; 3 Institute for Health Policy, Colombo, Sri Lanka; 4 The World Health Organization, Geneva, Switzerland

**Correction to: BMC Health Services Research (2024) 24:127**

10.1186/s12913-023-10464-0

In this article, the third author, Charlotte Dieteren, was omitted from the author group due to a typesetting mistake.

The author group has been updated above and the original article has been corrected. The publisher apologises to the authors and readers for the inconvenience caused by this error.

