# Supplementary material for: Health seeking behaviours and private sector delivery of care for non-communicable diseases in low- and middle-income countries: a systematic review
Source: BMC Health Serv Res. 2024 Jan 23;24:127. doi: 10.1186/s12913-023-10464-0 (PMC10807218; doi:10.1186/s12913-023-10464-0)
Supplement: Supplementary file 1 — Additional file 1. [file 12913_2023_10464_MOESM1_ESM.docx]

**Health seeking behaviours and private sector delivery of care for non-communicable diseases in low- and middle-income countries: a systematic review**

# Supplementary Material

Text S. 1 – Search strategy and selection process

Table S. 1 – Studies included in systematic review

Table S. 2 – Research designs & Mixed Methods Appraisal Tool (MMAT) ratings of selected studies

Table S. 3 – PRISMA Checklist

Text S. 1 – Extended description of the search strategy and selection process

Our search strategy was developed with the assistance of an information specialist, and conducted using Embase, Medline, Web of Science, Econlit and Global Index Medicus. The search was developed in Embase, optimized for sensitivity, then translated to other databases. The search strategies for Embase and Medline used relevant thesaurus terms from Emtree and Medical Subject Headings (MeSH) respectively. In all databases, the terms were searched in title, author keywords and major index terms; the search element for setting (health care providers in LMICs) was also searched in abstracts. We additionally searched Google Scholar and downloaded the 200 most relevant references in line with common practice.^1^ Our key terms were grouped into search elements: 1) non communicable diseases, 2) health care providers in LMICs, and 3) the determinants and outcomes of health seeking behaviour. The full search strategies for all databases are provided in the protocol registered in PROSPERO ([CRD42022340059](https://www.crd.york.ac.uk/PROSPERO/display_record.php?RecordID=340059)). After searching all databases, references were imported into EndNote and duplicates removed.^2^ We did not contact authors or subject experts.

| Search element | Key terms |
| --- | --- |
| What: noncommunicable diseases | noncommunicable diseases; cardiovascular disease; heart attack; stroke; myocardial infarction; hypertension; diabetes; cancer (top 5 for each sex: lung, breast, colorectum, prostate, stomach, liver, cervix, thyroid); chronic respiratory disease; chronic obstructive pulmonary disease; asthma |
| Where: health care providers in low- and middle-income countries | hospital, health clinic, community health centre, health centre, health post, family practitioner, general practitioner, primary care, secondary care, tertiary care, specialist, home care, nursing home, pharmacy, public provider, private provider, non-profit provider, faith-based provider, low-income countries, middle-income countries, lower-middle income countries, upper-middle income countries, list of all LMIC countries |
| Why and what happens: determinants and outcomes of health care decisions / health seeking behaviour | quality; cost; affordability; accessibility; proximity; distance; opening hours; in-network provider; reimbursement; reputation; health service delivery; health outcome; utilization; out-of-pocket payment; indirect costs; catastrophic health care expenditure; debt; health inequalities; health information; supply; fee for service; empowerment; patient reported experience |

The articles resulting from the search were screened by a team of two pairs (CB-JB & NW-CD) using Rayyan Reference Manager. To ensure harmonization, both teams started the selection process with a pilot review. Afterwards, each article was title-abstract screened independently by both individuals of one team using the eligibility criteria. To reduce bias, author names were not visible during screening.

Studies adhering to all eligibility criteria were selected for full-text review and title-abstract screened. If an inclusion/exclusion decision could not be made based on the title and abstract, the article was advanced to full-text screening. In the cases of a conflicting inclusion/exclusion decision, the other pair also reviewed the article, and the supervising researcher (IB) provided a final adjudication if necessary. Articles selected for full-text screening were reviewed by two individuals and the reasons for exclusion decisions were documented. Studies confirmed for inclusion were advanced to the next stage i.e. data extraction.

References

1. Harzing, A.W. (2007) Publish or Perish, <https://harzing.com/resources/publish-or-perish>
2. Bramer, W. M., Giustini, D., de Jonge, G. B., Holland, L. & Bekhuis, T. De-duplication of database search results for systematic reviews in EndNote*. J Med Libr Assoc* **104**, 240–243 (2016).

Table S. 1 – Studies included in systematic review

| Author/s | Year | Title | Journal | Country | Disease | Provider | Care | Research design | Sample | Data Source |
| --- | --- | --- | --- | --- | --- | --- | --- | --- | --- | --- |
| Adeniji | 2021 | [Burden of out-of-pocket payments among patients with cardiovascular disease in public and private hospitals in Ibadan, South West, Nigeria: A cross-sectional study](http://dx.doi.org/10.1136/bmjopen-2020-044044) | BMJ Open | Nigeria | Cardiovascular disease | Hospitals (Public & Private) | Curative & Rehabilitative | Cross-sectional | 744 patients who accessed healthcare | Survey data , 2019/2020 |
| Afkar et al. | 2021 | [Cost analysis of breast cancer: a comparison between private and public hospitals in Iran](http://dx.doi.org/10.1186/s12913-021-06136-6) | BMC Health Serv Res | Iran | Cancer (breast) | Hospitals (Public & Private) | Curative & Rehabilitative | Cross-sectional | 179 patients using care at private or public hopitals | Medical records and surveys, 2016/2017 |
| Akinyemiju | 2012 | [Socio-economic and health access determinants of breast and cervical cancer screening in low-income countries: analysis of the World Health Survey](https://doi.org/10.1371/journal.pone.0048834) | PLoS One | Multi-country (15) - Bangladesh, Burkina Faso, Chad, Comoros, Congo, Ethiopia, Ghana, Kenya, Laos, Malawi, Mali, Mauritania, Myanmar, Nepal, and Zimbabwe | Cancer (2) - breast and cervical | Clinics (Public, Private & NGO\|Other) | Prevention & Screening | Cross-sectional | 10,021 women aged 18- 69 for cervical cancer screening, and 4009 women aged 40-69 for breast cancer screening. | Survey data from WHS, 2003 |
| Banerjee et al. | 2022 | [Kolkata-Coventry comparative registry study of acute heart failure: an insight into the impact of public, private and universal health systems on patient outcomes in low-middle income cities (KOLCOV HF Study)](http://dx.doi.org/10.1136/openhrt-2022-001964%20) | Open Heart | India | Cardiovascular disease (heart failure) | Hospitals (Public & Private) | Chronic care management | Cohort (prospective) | 508 patients in private facility, 302 in public facility, 842 in UK facility. | Hospital survey data, 2014-2016 |
| Barakat et al. | 2018 | [Access to asthma medicines at the household level in eight counties of Kenya](http://dx.doi.org/10.5588/ijtld.17.0664) | Int J Tuberc Lung Dis | Kenya | Chronic respiratory disease (Asthma) | Hospitals (Public & Private) / Clinics (Public, Private & NGO\|Other) / Pharmacies (Public & Private) | Diagnosis / Medication | Cross-sectional | 128 asthma patients | Surveys |
| Barros et al. | 2019 | [Clinical pathways of breast cancer patients treated in the Federal District, Brazil](http://dx.doi.org/10.11606/S1518-8787.2019053000406) | Rev Saude Publica | Brazil | Cancer (breast) | Clinics (Public & Private) / Hospitals (Public & Private) | Diagnosis / Curative & Rehabilitative | Cross-sectional | 600 patients using care at nine public hospitals | Interviews, 2012/2014 |
| Bhojani et al. | 2013 | [Constraints faced by urban poor in managing diabetes care: patients' perspectives from South India](http://dx.doi.org/10.3402/gha.v6i0.22258) | Glob Health Action | India | Diabetes | Clinics (Public & Private) / Pharmacies (Public & Private) | Chronic care management /  Medicines | Phenomenology | 16 diabetes patients from a poor urban neighbourhood in South India | Interviews |
| Bhoo-Pathy et al. | 2019 | [Financial Toxicity After Cancer in a Setting With Universal Health Coverage: A Call for Urgent Action](https://doi.org/10.1200/JOP.18.00619) | J Oncol Pract | Malaysia | Cancer | Hospitals (Public & Private) | Curative & Rehabilitative | Cohort (prospective) | 1294 newly diagnosed patients with cancer using care at public or private hospitals | Cost diaries and surveys |
| Bigdeli et al. | 2016 | [Access to Treatment for Diabetes and Hypertension in Rural Cambodia: Performance of Existing Social Health Protection Schemes](https://doi.org/10.1371/journal.pone.0146147) | PLoS One | Cambodia | Multiple NCDs (2) - diabetes and hypertension | Clinics (Public & Private) / Pharmacies (Public & Private) | Diagnosis / Chronic care management / Medicines | Cross-sectional | 709 patients | Surveys, 2013 |
| Biswas et al. | 2017 | [Influence of distance between residence and health facilities on non-communicable diseases: An assessment over hypertension and diabetes in Bangladesh](http://dx.doi.org/10.1371/journal.pone.0177027) | PLoS ONE | Bangladesh | Multiple NCDs (2) - diabetes and hypertension | Clinics (Public & Private) / Pharmacies (Public & Private) | Diagnosis / Chronic care management | Cross-sectional | 7544 individuals aged 35 or older | Survey data from DHS, 2011 |
| Borges et al. | 2022 | [Publicly versus privately funded cardiac rehabilitation: access and adherence barriers. A cross-sectional study](http://dx.doi.org/10.1590/1516-3180.2020.0782.R1.31052021) | Sao Paulo Med J | Brazil | Cardiovascular disease | Clinics (Public & Private) | Chronic care management | Cross-sectional | 74 patients in public facility, 100 patients in private facility | Survey |
| Brinda et al. | 2012 | [Nature and determinants of out-of-pocket health expenditure among older people in a rural Indian community](https://doi.org/10.1017/S104161021200083X) | Int. Psychogeriatr. | India | Diabetes | Clinics (Public & Private) / Pharmacies (Public & Private) | Chronic care management /  Medicines | Cross-sectional | 1000 individuals aged 60+ and living in South India | Surveys, 2004/2005 |
| Brinda et al. | 2015 | [Health service use, out-of-pocket payments and catastrophic health expenditure among older people in India: The WHO Study on global AGEing and adult health â€¦](https://doi.org/10.1136/jech-2014-204960) | J Epidemiol Community | India | Multiple NCDs (4) - Cardiovascular disease, chronic obstructive pulmonary disease, diabetes, hypertension | Clinics (Public & Private) / Pharmacies (Public & Private) | Chronic care management /  Medicines | Cross-sectional | 2414 people aged 65+ | Survey data from SAGE, 2007/2008 |
| Brito-Silva et al. | 2014 | [Integrality in cervical cancer care: evaluation of access](https://doi.org/10.1590/S0034-8910.2014048004852) | Rev Saude Publica | Brazil | Cancer (cervical) | Clinics (Public & Private) / Hospitals (Public & Private) | Diagnosis / Curative & Rehabilitative | Cross-sectional / Phenomenology | A. 146,868 women aged 25-29 and recorded in the health information system. B. 10 healthcare professionals, 2 representatives of municipal administration and 10 users. | A. Medical records, 2008 - 2010. B. interviews |
| Brown et al. | 2014 | [Use of medical services and medicines attributable to diabetes in sub-saharan Africa](http://dx.doi.org/10.1371/journal.pone.0106716) | PLoS ONE | Multi-country (4) - Cameroon, Mali, South Africa, and Tanzania | Diabetes | Clinics (Public & Private) / Hospitals (Public & Private) | Diagnosis / Chronic care management / Medicines | Cross-sectional | 1780 persons with diagnosed diabetes using care, 1780 matched comparison subjects | Surveys, 2008/2009 |
| Burrowes et al. | 2022 | [Perceptions of cervical cancer care among Ethiopian women and their providers: a qualitative study](http://dx.doi.org/10.1186/s12978-021-01316-3) | Reprod Health | Ethiopia | Cancer (cervical) | Clinics (Public & Private) / Hospitals (Public & Private) | Diagnosis / Curative & Rehabilitative | Narrative inquiry | 15 patients, 30 healthcare providers | Interviews, 2017 |
| Caeiro et al. | 2020 | [Type 2 diabetes. Prevalence of hypoglycemia in public versus private health care system](https://pubmed.ncbi.nlm.nih.gov/32442934/) | Medicina (B Aires) | Argentina | Diabetes | Clinics (Public & Private) / Hospitals (Public & Private) | Diagnosis / Curative & Rehabilitative | Cross-sectional | 600 adult patients using care at public or private care facilities | Surveys, 2017/2018 |
| Chakraborty et al. | 2022 | [Health care seeking behaviour and financial protection of patients with hypertension: A cross-sectional study in rural West Bengal, India](http://dx.doi.org/10.1371/journal.pone.0264314) | PLoS ONE | India | Hypertension | Clinics (Public & Private) | Chronic care management | Cross-sectional | 300 people with hypertension | Survey, 2017-2018 (3 month period) |
| Chimberengwa et al. | 2019 | [Knowledge, attitudes and practices related to hypertension among residents of a disadvantaged rural community in southern Zimbabwe](https://doi.org/10.1371/journal.pone.0215500) | PloS one | Zimbabwe | Hypertension | Clinics (Public & Private) | Diagnosis / Chronic care management | Cross-sectional | 304 adult patients who were residing in a rural area | Survey |
| Clarke-Deelder et al. | 2022 | [Health care seeking in modern urban LMIC settings: evidence from Lusaka, Zambia](https://doi.org/10.1186/s12889-022-13549-3) | BMC Public Health | Zambia | Chronic NCDs (all) | Clinics (Public & Private) / Hospitals (Public & Private) | Diagnosis / Curative & Rehabilitative / Chronic care management | Cross-sectional | 620 housholds linked to 88 health facilities | Interviews, 2020 |
| Coutinho Medeiros et al. | 2019 | [Factors influencing delay in symptomatic presentation of breast cancer in Brazilian women](https://doi.org/10.1111/hsc.12823) | Health Soc Care Community | Brazil | Cancer (breast) | Clinics (Public & Private) / Hospitals (Public & Private) | Diagnosis | Cohort (prospective) | 388 patients admitted to a public hospital | Surveys, 2014/2015 |
| Cremers et al. | 2019 | [Patients' and healthcare providers' perceptions and practices regarding hypertension, pharmacy-based care, and mHealth in Lagos, Nigeria: a mixed methods study](http://dx.doi.org/10.1097/hjh.0000000000001877) | J Hypertens | Nigeria | Hypertension | Clinics (Public & Private) / Pharmacies (Public & Private) / mHealth | Chronic care management /  Medicines | Cross-sectional / Phenomenology | A. 30 patients, 9 pharmacists, 6 cardiologists, 1 chairman of the ACPN, B. patients and pharmacists C. 9 pharmacies, D. 328 patients | A. in-depth interviews. B. Focus Groups. C. Observations .D. baseline interviews |
| de Castro Figueiredo Pereira Coelho et al. | 2018 | [Impact on the quality of life of women with breast cancer undergoing chemotherapy in public and private care](http://dx.doi.org/10.17533/udea.iee.v36n1e04) | Investigacion Educ Enferm | Brazil | Cancer (breast) | Hospitals (Public & Private) | Curative & Rehabilitative | Cohort (prospective) | 115 female patients using public or private care | Surveys, 2012-2015 |
| de Melo Santos et al. | 2020 | [Availability of Diagnostic Services and their Impact on Patient Flow in Two Brazilian Referral Centres of Breast Cancer Treatment](http://dx.doi.org/10.31557/apjcp.2020.21.2.317) | Asian Pac J Cancer Prev | Brazil | Cancer (breast) | Clinics (Public & Private) / Hospitals (Public & Private) | Diagnosis / Curative & Rehabilitative | Cohort (prospective) | 128 patients who received treatment in specific referral centres | Medical records, 2016 |
| Deek et al. | 2020 | [The Lebanese Heart Failure Snapshot: A National Presentation of Acute Heart Failure Admissions](https://doi.org/10.1111/jnu.12583) | J Nurs Scholarsh | Lebanon | Cardiovascular disease (heart failure) | Hospitals (Public & Private) | Curative & Rehabilitative | Cohort (prospective) | 137 adult patients presenting to one of the study sites with symptoms | Surveys, 2019 |
| Durán et al. | 2018 | [Sharp rise in the expenditures of targeted drugs in Ecuador: five-year (2010â€“2014) consumption of oncologic drugs in public and private hospitals](http://dx.doi.org/10.1111/jphs.12221) | J Pharm Health Serv Res | Ecuador | Cancer | Hospitals (Public & Private) | Medicines | Quasi experiment | 40,099 patients based on use of oncologic drug | Medical records, 2010-2014 |
| Elias et al. | 2017 | [Preparedness for delivering non-communicable disease services in primary care: access to medicines for diabetes and hypertension in a district in south India](http://dx.doi.org/10.1136/bmjgh-2017-000519) | BMJ glob. health | India | Multiple NCDs (2) - diabetes and hypertension | Clinics (Public & Private) / Pharmacies (Public & Private) | Chronic care management /  Medicines | Cross-sectional / Phenomenology | A. 1149 patients, B. 39 primary health centers and 30 private pharmacies, C. community members, health workers, PHC medical officers and pharmacists from private pharmacies, D. PHC medical offers and district health officials. | A. Surveys, B. Facility survey, C. Focus groups, D. Interviews |
| Emmerick et al. | 2017 | [Retrospective interrupted time series examining hypertension and diabetes medicines usage following changes in patient cost sharing in the 'Farmacia Popular' programme in Brazil](http://dx.doi.org/10.1136/bmjopen-2017-017308) | BMJ Open | Brazil | Multiple NCDs (2) - diabetes and hypertension | Pharmacies (Public & Private) | Medicines | Quasi experiment | 6,032,380 and 14,392,076 patients who used oral hypoglycaemic or antihypertensive medicines | Pharmacy records, 2008-2012 |
| Eshwari et al. | 2021 | [Economic burden of type 2 diabetes mellitus management: Epidemiological determinants from a coastal community of Southern India](http://dx.doi.org/10.4103/WHO-SEAJPH.WHO-SEAJPH_20_21%20) | WHO South East Asia J Public Health | India | Diabetes | Hospitals (Public & Private) / Clinics (Public, Private & NGO\|Other) / Pharmacies (Public & Private) | Chronic care management | Cross-sectional | 809 participants seeking care for chronic management of diabetes | Survey, 2012-2015 |
| Fayehun et al. | 2022 | [A contextual exploration of healthcare service use in urban slums in Nigeria](https://doi.org/10.1371/journal.pone.0264725) | PloS ONE | Nigeria | Chronic NCDs (all) | Hospitals (Public & Private) / Clinics (Public, Private & NGO\|Other) / Pharmacies (Public & Private) | Diagnosis / Curative & Rehabilitative | Cross-sectional | 1634 residents of slums in Sothwestern Nigeria | Survey |
| Fayyaz et al. | 2020 | [The quality of patient care in oncology departments in Karachi, Pakistan: patients' perceptions](https://doi.org/10.1108/IJHCQA-12-2019-0201) | Int J Health Care Qual Assur | Pakistan | Cancer | Hospitals (Public & Private) | Curative & Rehabilitative | Cross-sectional | 389 adult patients using care at public or private hospitals | Surveys, 2018 |
| Fernandopulle et al. | 2019 | [Patient experiences of access to NCD medicines in Sri Lanka: Evidence of the success story towards universal coverage](http://dx.doi.org/10.1177/2399202619873228) | Med Access Point Care | Sri Lanka | Multiple NCDs (3) - chronic respiritory disease (asthma and chronic obstructive pulmonary disease), cardiovascular disease (ischemic heart disease), diabetes | Pharmacies (Public & Private) | Medicines | Cross-sectional | 1008 patients aged 50+ | Surveys, 2018 |
| Franken et al. | 2020 | [Performance of acute coronary syndrome approaches in Brazil: A report from the BRACE (Brazilian Registry in Acute Coronary SyndromEs)](http://dx.doi.org/10.1093/ehjqcco/qcz045) | Eur Heart J Qual Care Clin Outcomes | Brazil | Cardiovascular disease | Hospitals (Public & Private) | Curative & Rehabilitative | Cross-sectional | 1150 adult patients | Registry data, 2007-2009 |
| Gabrani et al. | 2022 | [Out of pocket payments and access to NCD medication in two regions in Albania](http://dx.doi.org/10.1371/journal.pone.0272221) | PLoS ONE | Albania | Chronic NCDs (all) | Hospitals (Public & Private) / Clinics (Public, Private & NGO\|Other) / Pharmacies (Public & Private) | Chronic care management | Cross-sectional | 898 residents of two Albanian regions who consulted a health care provider for a chronic health condition in past 8 weeks | Survey, 2018 |
| Gonzélez-Villalpando et al. | 2010 | [The status of diabetes care in Mexican population: are we making a difference? Results of the National Health and Nutrition Survey 2006](https://doi.org/10.1590/S0036-36342010000700007) | Salud Publica Mex | Mexico | Diabetes | Clinics (Public & Private) / Hospitals (Public & Private) | Diagnosis / Chronic care management | Cross-sectional | 2644 adult patients | Survey data from National Health and Nutrition Survey, 2006 |
| Gyenwali et al. | 2013 | [Factors associated with late diagnosis of cervical cancer in Nepal](https://doi.org/10.7314/APJCP.2013.14.7.4373) | Asian Pac J Cancer Prev | Nepal | Cancer (cervical) | Clinics (Public & Private) / Hospitals (Public & Private) | Diagnosis | Cross-sectional | 110 first-time patients using hospital care | Surveys and medical records, 2012 |
| Gyenwali et al. | 2014 | [Estimates of delays in diagnosis of cervical cancer in Nepal](http://dx.doi.org/10.1186/1472-6874-14-29) | BMC Womens Health | Nepal | Cancer (cervical) | Clinics (Public & Private) / Hospitals (Public & Private) | Diagnosis | Cross-sectional | 110 first-time patients using hospital care | Surveys and medical records, 2012 |
| Hagos et al. | 2020 | [Economic burden and predictors of cost variability among adult cancer patients at comprehensive specialized hospitals in West Amhara, Northwest Ethiopia, 2019](http://dx.doi.org/10.2147/CMAR.S282746) | Cancer Manage Res | Ethiopia | Cancer | Hospitals (Public & Private) | Curative & Rehabilitative | Cross-sectional | 464 adult patients using hospital care (public or teaching hospital) | Surveys, 2019 |
| Hassan et al. | 2015 | [A cross sectional study on the motivators for Asian women to attend opportunistic mammography screening in a private hospital in Malaysia: the MyMammo study](http://dx.doi.org/10.1186/s12889-015-1892-1) | BMC Public Health | Malaysia | Cancer (breast) | Hospital (Private) | Prevention & Screening | Cross-sectional | 1453 women aged 40-74, attending an opportunistic screening program at a private hospital | Surveys, 2011-2013 |
| Herbst et al. | 2020 | [Colorectal Cancer (CRC) treatment and associated costs in the public sector compared to the private sector in Johannesburg, South Africa](http://dx.doi.org/10.1186/s12913-020-05112-w) | BMC Health Serv Res | South Africa | Cancer (colorectal) | Hospital (Private) | Curative & Rehabilitative | Cross-sectional | 729 newly diagnosed patients with a private medical scheme | Administrative claim data, 2012-2015 |
| Herskind et al. | 2020 | [The outpatient management of hypertension at two Sierra Leonean health centres: A mixed-method investigation of follow-up compliance and patient-reported barriers to care](http://dx.doi.org/10.4102/phcfm.v12i1.2222) | Afr J Prim Health Care Fam Med | Sierra Leone | Hypertension | Clinics (Private) | Chronic care management /  Medicines | Cross-sectional / Phenomenology | A. 487 adult patients, B. 68 adult patients | A. Medical records and B. surveys, 2017/2018 |
| Higuchi | 2010 | [Access to diabetes care and medicines in the Philippines](https://doi.org/10.1177/1010539510373005) | Asia Pac J Public Health | Philippines | Diabetes | Clinics (Public & Private) / Pharmacies (Public & Private) | Chronic care management /  Medicines | Cross-sectional / Phenomenology | 359 individuals from different health care settings | Surveys |
| Ide et al. | 2018 | [Barriers and facilitators of diabetes services in Nepal: a qualitative evaluation](http://dx.doi.org/10.1093/heapol/czy011) | Health Policy Plan | Nepal | Diabetes | Clinics (Public & Private) / Hospitals (Public & Private) | Chronic care management /  Medicines | Narrative inquiry | A. 44 patients for > 1 year attending one of the study sites and B. 46 community members without diabetes | A. Interviews and B. focus groups, 2015/2016 |
| Jalilian et al. | 2019 | [Health care seeking behaviors in type 2 diabetic patients in East Azerbaijan](http://dx.doi.org/10.5603/DK.2019.0031) | Clin Diabetol | Azerbaijan | Diabetes | Clinics (Public & Private) / Hospitals (Public & Private) | Chronic care management /  Medicines | Cross-sectional | 1200 adult patients using care | Surveys |
| Jankovic et al. | 2019 | [Patterns of Health Care Utilization for Noncommunicable Diseases in a Transitional European Country: Results from the National Health Survey](https://doi.org/10.1177/0020731418762717) | Int J Health Serv | Bosnia and Herzegovina | Chronic NCDs (all) | Clinics (Public & Private) / Hospitals (Public & Private) | Chronic care management | Cross-sectional | 4128 adults | Survey data from National Health Survey, 2010 |
| Jayanna et al. | 2019 | [Designing a comprehensive Non-Communicable Diseases (NCD) programme for hypertension and diabetes at primary health care level: evidence and experience from urban Karnataka, South India](http://dx.doi.org/10.1186/s12889-019-6735-z) | BMC Public Health | India | Multiple NCDs (2) - diabetes and hypertension | Clinics (Public & Private) | Chronic care management | Cross-sectional / Phenomenology | A. 157 adults patients urban primary health care, B: 20 patients, C. 10 patients | A. Surveys, B. interviews and C. focus groups, 2017/2018 |
| Jeyashree et al. | 2017 | [Inequity in access to inpatient healthcare services for non-communicable diseases in India and the role of out-of-pocket payments](https://doi.org/10.4103/0970-258X.234390) | Natl Med J India | India | Multiple NCDs (6) - chronic respiritory disease (asthma), cancers, cardiovacular disease, diabetes, hypertension, neurological and psychiatric disorders | Clinics (Public & Private) | Chronic care management /  Medicines | Cross-sectional | 4392 patients hospitalized for specified NCDs | Survey data from National Sample Survey, 2004/2005 |
| Jiamjarasrangsi et al. | 2014 | [Assessment of 5-year system-wide type 2 diabetes control measures in a Southeast Asian metropolis](http://dx.doi.org/10.5372/1905-7415.0801.264) | Asian Biomed | Thailand | Diabetes | Clinics (Public & Private) / Hospitals (Public & Private) | Chronic care management | Cross-sectional | 1000 adult patients attending public or private care facilities | Surveys and medical records, 2011/2012 |
| Jimeno et al. | 2021 | [Direct medical costs of type 2 diabetes mellitus in the Philippines: findings from two hospital databases and physician surveys](https://doi.org/10.1136/bmjopen-2021-049737) | BMJ Open | Philippines | Diabetes | Hospitals (Public & Private) | Curative & Rehabilitative / Chronic care management | Cross-sectional | 1023 and 1378 adult patients attending a teaching or specialised hospital which includes private beds | Medical records, 2016 |
| Julião et al. | 2021 | [Trends in the prevalence of systemic arterial hypertension and health care service use in Brazil over a decade (2008-2019)](http://dx.doi.org/10.1590/1413-81232021269.08092021) | Cien Saude Colet | Brazil | Hypertension | Clinics (Public & Private) | Diagnosis / Chronic care management | Cross-sectional | 60,202 and 88,531 adult individuals | Survey data from PNS survey, 2013 & 2019 |
| Karinja et al. | 2019 | [Care-seeking dynamics among patients with diabetes mellitus and hypertension in selected rural settings in Kenya](https://doi.org/10.3390/ijerph16112016) | International journal of â€¦ | Kenya | Multiple NCDs (2) - diabetes and hypertension | Clinics (Public & Private) / Pharmacies (Public & Private) | Chronic care management /  Medicines | Cross-sectional | 1,100 adult patients in rural areas | Surveys, 2016/2017 |
| Kastor et al. | 2018 | [Disease and age pattern of hospitalisation and associated costs in India: 1995-2014](https://doi.org/10.1136/bmjopen-2017-016990) | BMJ Open | India | Multiple NCDs (4) - cancer, cardiovascular disease (heart disease), diabetes, hypertension | Hospitals (Public & Private) | Curative & Rehabilitative | Cross-sectional | 633,408 individuals (1995) and 335,499 individuals (2014) | Survey data from National Sample Survey, 1995 & 2004 |
| Kathrikolly et al. | 2020 | [Opportunities and Barriers to Breast Cancer Screening in a Rural Community in Coastal Karnataka, India: A Qualitative Analysis](http://dx.doi.org/10.31557/APJCP.2020.21.9.2569) | Asian Pac J Cancer Prev | India | Cancer (breast) | Clinics (Public & Private) | Prevention & Screening | Phenomenology | 44 women aged 20-60 years, from low and middle-income households | Focus groups, 2015/2019 |
| Kiragu et al. | 2022 | [Household access to non-communicable disease medicines during universal health care roll-out in Kenya: A time series analysis](http://dx.doi.org/10.1371/journal.pone.0266715) | PLoS ONE | Kenya | Chronic NCDs (all) | Clinics (Public) / Hospitals (Public/Private) / Pharmacies (Private) | Medicines | Randomised control trial | 385 individuals | Telephone interviews, 2016-2019 |
| Kohler et al. | 2017 | [A framework for improving early detection of breast cancer in sub-Saharan Africa: A qualitative study of help-seeking behaviors among Malawian women](http://dx.doi.org/10.1016/j.pec.2016.08.012) | Patient Educ Couns | Malawi | Cancer (breast) | Clinics (Public & Private) / Hospitals (Public & Private) | Prevention & Screening | Grounded theory | 20 patients using care at teaching hospital | Interviews, 2014 |
| Kong et al. | 2017 | [Advanced Stage at Presentation Remains a Major Factor Contributing to Breast Cancer Survival Disparity between Public and Private Hospitals in a Middle-Income Country](http://dx.doi.org/10.3390/ijerph14040427) | IJERPH | Malaysia | Cancer (breast) | Hospitals (Public & Private) | Curative & Rehabilitative | Cohort (prospective) | 3966 newly diagnosed patients | Registry data, 2001-2011 |
| Kujawski et al. | 2018 | [Reasons for low utilisation of public facilities among households with hypertension: analysis of a population-based survey in India](http://dx.doi.org/10.1136/bmjgh-2018-001002) | BMJ glob. health | India | Multiple NCDs (2) - diabetes and hypertension | Clinics (Public & Private) / Hospitals (Public & Private) | Chronic care management /  Medicines | Cross-sectional | 336,305 households | Survey data from District Level Household and Facility Survey, 2012/2013 |
| Kumar et al. | 2019 | [Delays in Diagnosis and Treatment of Breast Cancer and the Pathways of Care: A Mixed Methods Study from a Tertiary Cancer Centre in North East India](http://dx.doi.org/10.31557/apjcp.2019.20.12.3711) | Asian Pac J Cancer Prev | India | Cancer (breast) | Clinics (Public & Private) / Hospitals (Public & Private) | Diagnosis / Curative & Rehabilitative | Cohort (retrospective) / Phenomenology | A. 469 patients using hospital care, B. 15 patients (and 10 HCP, results not extracted) | A. Medical records and survey, B. Interviews, 2019 |
| Kumara et al. | 2017 | [Impact of ill-health on household consumption in Sri Lanka: Evidence from household survey data](https://doi.org/10.1016/j.socscimed.2017.11.015) | Soc Sci Med | Sri Lanka | Chronic NCDs (all) | Hospitals (Public & Private) | Chronic care management /  Medicines | Cross-sectional | 20,535 households | Survey data from Sri Lanka Household Income and Expenditure Survey, 2012/2013 |
| Lall et al. | 2019 | [Challenges in primary care for diabetes and hypertension: an observational study of the Kolar district in rural India](https://doi.org/10.1186/s12913-019-3876-9) | BMC health services â€¦ | India | Multiple NCDs (2) - diabetes and hypertension | Clinics (Public & Private) | Curative & Rehabilitative / Chronic care management | Cross-sectional / Phenomenology | 24 patients seeking care in private and public facilities and 6 care providers | Interviews and observations, 2016/2017 |
| Lee et al. | 2022 | [Hypertension awareness, treatment, and control and their association with healthcare access in the middle-aged and older Indian population: A nationwide cohort study](http://dx.doi.org/10.1371/journal.pmed.1003855) | PLoS Med | India | Multiple NCDs (2) - cardiovascular disease and hypertension | Clinics (Public & Private) | Diagnosis / Chronic care management | Cohort (prospective) | 1683 and 72,262 individuals aged 40+ | Survey data from LASI, 2010 & 2017/2018 |
| Legido-Quigley et al. | 2019 | [Patients' experiences on accessing health care services for management of hypertension in rural Bangladesh, Pakistan and Sri Lanka: A qualitative study](https://doi.org/10.1371/journal.pone.0211100) | PLoS One | Multi-country (3) - Bangladesh, Pakistan, Sri Lanka | Hypertension | Clinics (Public & Private) | Chronic care management | Narrative inquiry | 60 patients living in rural areas | Interviews, 2016/2017 |
| Lopes Ibanez-Gonzalez et al. | 2013 | [Chronic non-communicable disease and healthcare access in middle-aged and older women living in Soweto, South Africa](https://doi.org/10.1371/journal.pone.0078800) | PLoS One | South Africa | Chronic NCDs (all) | Clinics (Public & Private) | Chronic care management | Cross-sectional | 1102 women aged 30+ | Surveys, 2008/2010 |
| Mahalakshmi et al. | 2020 | [Barriers to Cancer Screening Uptake in Women: A Qualitative Study from Tamil Nadu, India](http://dx.doi.org/10.31557/apjcp.2020.21.4.1081) | Asian Pac J Cancer Prev | India | Cancer (2) - breast and cervical | Clinics (Public & Private) / Hospitals (Public & Private) | Prevention & Screening | Narrative inquiry | 10 women and 9 service providers | Interviews, 2019 |
| Malta et al. | 2022 | [Self-reported arterial hypertension, use of health services and guidelines for care in Brazilian population: National Health Survey, 2019](http://doi.org/10.1590/SS2237-9622202200012.especial) | Epidemiol. serv. saude | Brazil | Hypertension | Clinics (Public/Private) / Pharmacies | Diagnosis / Curative & Rehabilitative / Chronic care management / Medicines | Cross-sectional | 88531 individuals in general public | Survey data from National Health Survey, 2019 |
| Medeiros et al. | 2021 | [Determinants of delay from cancer diagnosis to treatment initiation in a cohort of brazilian women with breast cancer](https://doi-org.eur.idm.oclc.org/10.1111/hsc.13284) | Health Soc Care Community | Brazil | Cancer (breast) | Hospitals (Public & Private) | Diagnosis / Curative & Rehabilitative | Cohort (prospective) | 470 patients at first hospital consultation | Surveys and medical records, 2014-2015 |
| Men et al. | 2012 | [I wish I had AIDS: a qualitative study on access to health care services for HIV/AIDS and diabetic patients in Cambodia](https://doi.org/10.5195/HCS.2012.67) | Health, Culture and Society | Cambodia | Diabetes | Clinics (Public & Private) / Hospitals (Public & Private) | Diagnosis / Chronic care management | Narrative inquiry | 25 HIV/AIDS and 45 diabetic patients | Interviews, 2005 |
| Mendenhall et al. | 2015 | [Diabetes care among urban women in Soweto, South Africa: a qualitative study](http://dx.doi.org/10.1186/s12889-015-2615-3) | BMC Public Health | South Africa | Diabetes | Clinics (Public & Private) / Pharmacies (Public & Private) | Chronic care management /  Medicines | Cross-sectional / Phenomenology | 27 female patients | Interviews, 2012 |
| Mendoza et al. | 2021 | [(De)constructing ‘therapeutic itineraries’ of hypertension care: A qualitative study in the Philippines](https://doi.org/10.1016/j.socscimed.2021.114570) | Soc Sci Med | Philippines | Cardiovascular disease | Hospitals (Public & Private) / Clinics (Public, Private & NGO\|Other) / Pharmacies (Public & Private) | Diagnosis / Chronic care management / Medicines | Grounded theory | 40 patients | Repeated interviews and diaries, 2018-2019 |
| Musinguzi et al. | 2018 | [Factors influencing compliance and health seeking behaviour for hypertension in Mukono and Buikwe in Uganda: a qualitative study](https://doi.org/10.1155/2018/8307591) | Int journal of hypertension | Uganda | Hypertension | Clinics (Public & Private) / Pharmacies (Public & Private) | Medicines | Phenomenology | 48 patients | Interviews, 2014 |
| Nang et al. | 2019 | [Patients' and healthcare providers' perspectives of diabetes management in Cambodia: a qualitative study](http://dx.doi.org/10.1136/bmjopen-2019-032578) | BMJ Open | Cambodia | Diabetes | Clinics (Public & Private) / Pharmacies (Public & Private) | Chronic care management /  Medicines | Cross-sectional / Phenomenology | 59 patients and 30 providers | Interviews and focus groups |
| Nardi et al. | 2012 | [Comparison of the epidemiologic features and patterns of initial care for prostate cancer between public and private institutions: a survey by the Brazilian Society of Urology](https://doi.org/10.1590/S1677-55382012000200003) | Int Braz J Urol | Brazil | Cancer (prostate) | Hospitals (Public & Private) | Diagnosis / Curative & Rehabilitative | Cross-sectional | 1915 patients attending care at public and private providers | Medical records, 2014-2015 |
| Naydenova et al. | 2017 | [Healthcare choices in Mumbai slums: A cross-sectional study](http://dx.doi.org/10.12688/wellcomeopenres.13127.2) | Wellcome Open Res | India | Multiple NCDs (2) - diabetes and hypertension | Clinics (Public & Private) / Pharmacies (Public & Private) | Diagnosis / Chronic care management / Medicines | Cross-sectional / Phenomenology | 549 slum dwellers | Surveys, 2015 |
| Ntombela et al. | 2017 | [Is the clinicopathological pattern of colorectal carcinoma similar in the state and private healthcare systems of South Africa? Analysis of a Durban colorectal cancer database](http://dx.doi.org/10.1177/0049475517710887) | Trop Doct | South Africa | Cancer (colorectal) | Hospitals (Public & Private) | Diagnosis / Curative & Rehabilitative | Cross-sectional | 419 patients diagnosed using public or private hospital care | Registry data, 2009 |
| Olaleye et al. | 2017 | [Satisfaction of Nigerian stroke survivors with outpatient physiotherapy care](https://doi.org/10.1590/S1677-55382012000200003) | Physiother Theory Pract | Nigeria | Cardiovascular disease (stroke) | Hospitals (Public & Private) / Clinics (Public, Private & NGO\|Other) | Rehabilitative | Phenomenology | 60 patients receiving out-patient care at public or private facilities | Surveys and focus-groups, 2013 |
| Oliveira et al. | 2019 | [Disparities in Access and Mortality of Patients With ST-Segmentâ€“Elevation Myocardial Infarction Using the Brazilian Public Healthcare System: VICTIM Register](http://dx.doi.org/10.1161/JAHA.119.013057) | J Am Heart Assoc | Brazil | Cardiovascular disease | Hospitals (Public & Private) | Curative & Rehabilitative | Cross-sectional | 707 patients using public or private care | Medical records, 2014-2017 |
| Onyango et al. | 2018 | [Perceptions of Kenyan adults on access to medicines for non-communicable diseases: A qualitative study](https://doi.org/10.1371/journal.pone.0201917) | PLoS One | Kenya | Chronic NCDs (all) | Clinics (Public & Private) / Pharmacies (Public & Private) | Medicines | Phenomenology | 84 patients who had been diagnosed and treated for an NCD | Surveys |
| Opare-Addo et al. | 2020 | [Healthcare services utilisation among patients with hypertension and diabetes in rural Ghana](http://dx.doi.org/10.4102/phcfm.v12i1.2114) | Afr J Prim Health Care Fam Med | Ghana | Multiple NCDs (2) - diabetes and hypertension | Clinics (Public & Private) / Hospitals (Public & Private) | Diagnosis / Chronic care management | Cross-sectional | 684 adults in rural districts | Surveys, 2016 |
| Ozdemir et al. | 2021 | [Five-Year Trends in Direct Costs of Chronic Obstructive Pulmonary Disease in Turkey: COPDTURKEY-3](https://doi.org/10.5152/TurkThoracJ.2021.19150) | Turk Thorac J | Turkey | Chronic respiritory disease (chronic obstructive pulmonary disease) | Hospitals (Public & Private) | Curative & Rehabilitative | Cross-sectional | 6,010,900 patients | Administrative healthcare data, 2012-2016 |
| Pati et al. | 2020 | [Magnitude and determinants of multimorbidity and health care utilization among patients attending public versus private primary care: a cross-sectional study â€¦](https://doi.org/10.1186/s12939-020-01170-y) | Int J Equity Health | India | Chronic NCDs (all) | Clinics (Public & Private) | Diagnosis / Chronic care management | Cross-sectional | 1649 patients using care at public or private primary care facilities | Surveys, 2013/2014 |
| Paz-Soldán et al. | 2012 | [Structural barriers to screening for and treatment of cervical cancer in Peru](http://dx.doi.org/10.1016/S0968-8080(12)40680-2) | Reprod Health Matters | Peru | Cancer (cervical) | Clinics (Public & Private) / Hospitals (Public & Private) | Prevention & Screening / Curative & Rehabilitative | Phenomenology | 30 informants from different institutions in the health care system | Interviews, 2007 |
| Perera et al. | 2019 | [Patient perspectives on hypertension management in health system of Sri Lanka: a qualitative study](https://doi.org/10.1136/bmjopen-2019-031773) | BMJ Open | Sri Lanka | Hypertension | Clinics (Public & Private) | Chronic care management | Narrative inquiry | 20 patients in rural areas | Interviews |
| Pinchevsky et al. | 2018 | [Quality of care delivered to type 2 diabetes mellitus patients in public and private sector facilities in Johannesburg, South Africa](http://dx.doi.org/10.2147/IJGM.S165545) | Int J Gen Med | South Africa | Diabetes | Clinics (Public & Private) | Chronic care management | Cross-sectional / Phenomenology | 290 adult patients using care at public or private facilities | Medical records and surveys, 2016 |
| Priya et al. | 2020 | [Patient's Experiences and Satisfaction in Diabetes Care and Out-of-Pocket Expenditure for Follow-Up Care Among Diabetes Patients in Urban Puducherry, South India](http://dx.doi.org/10.1177/2374373519898919) | J Patient Exp | India | Diabetes | Hospitals (Public & Private) | Curative & Rehabilitative | Cross-sectional | 200 patients for >1 year using care at public or private facility | Surveys, 2016 |
| Rahmawati et al. | 2018 | [Access to medicines for hypertension: a survey in rural Yogyakarta province, Indonesia](https://doi.org/10.22605/RRH4393) | Rural Remote Health | Indonesia | Hypertension | Clinics (Public & Private) / Pharmacies (Public & Private) | Medicines | Cross-sectional | 384 patients aged 45+ in rural villages | Surveys, 2015 |
| Rajasulochana et al. | 2021 | [Economic burden associated with stroke in India: insights from national sample survey 2017-18](https://doi.org/10.1080/14737167.2021.1941883) | Expert Rev Pharmacoecon Outcomes Res | India | Cardiovascular disease (stroke) | Hospitals (Public & Private) | Curative & Rehabilitative | Cross-sectional | 1152 hospitalized patients and 407 patients using out patient care | Survey data from National Sample Survey Organization, 2017/2018 |
| Rangel-Mendez et al. | 2018 | [Healthcare delay in breast cancer patients: a case study in a low-density population region from Mexico](http://dx.doi.org/10.2217/fon-2017-0713) | Fut Oncol | Mexico | Cancer (breast) | Hospitals (Public & Private) | Diagnosis / Curative & Rehabilitative | Cohort (retrospective) | 92 patients at tertiary care hospital | Medical records, 2010-2012 |
| Rannan-Eliya et al. | 2015 | [The quality of outpatient primary care in public and private sectors in Sri Lanka--how well do patient perceptions match reality and what are the implications?](http://dx.doi.org/10.1093/heapol/czu115) | Health Policy Plan | Sri Lanka | Multiple NCDs (4) - chronic respiritory disease (asthma), hypertension, hypercholesterolemia, diabetes | Clinics (Public & Private) / Hospitals (Public & Private) | Diagnosis / Curative & Rehabilitative / Chronic care management | Cross-sectional | 1027 public sector patients and 944 private sector patients using outpatients care | Observations and interviews, 2012 |
| Rannan-Eliya et al. | 2015 | [Quality of inpatient care in public and private hospitals in Sri Lanka](http://dx.doi.org/10.1093/heapol/czu062) | Health Policy Plan | Sri Lanka | Multiple NCDs (2) - chronic respiritory disease (asthma and chronic obstructive pulmonary disease), cardiovascular disease (acute myocardial infarction and stroke) | Clinics (Public & Private) / Hospitals (Public & Private) | Diagnosis / Curative & Rehabilitative / Chronic care management | Cross-sectional | 2523 and 1815 patients using care at public or private hospitals | Medical records, 2011 |
| Rao et al. | 2011 | [Socio-economic inequalities in the financing of cardiovascular & diabetes inpatient treatment in India](https://pubmed.ncbi.nlm.nih.gov/21321420/) | Indian J Med Res | India | Multiple NCDs (2) - cardiovascular disease and diabetes | Hospitals (Public & Private) | Curative & Rehabilitative | Cross-sectional | 2129 hospitalized CVD patients and 438 hospitalised diabetes patients | Survey data from National Sample Survey Organization, 2004 |
| Recondo et al. | 2019 | [Access of patients with breast and lung cancer to chemotherapy treatment in public and private hospitals in the city of Buenos Aires](http://dx.doi.org/10.1093/intqhc/mzz047) | Int J Qual Health Care | Argentina | Cancer (2) - breast and lung | Hospitals (Public & Private) | Diagnosis / Curative & Rehabilitative | Cohort (retrospective) | 168 adult patients with breast cancer and or 100 adult patients with lung cancer treated with chemotherapy in public or private setting | Medical records and surveys, 2016 |
| Risso-Gill et al. | 2015 | [Understanding the modifiable health systems barriers to hypertension management in Malaysia: a multi-method health systems appraisal approach](http://dx.doi.org/10.1186/s12913-015-0916-y) | BMC Health Serv Res | Malaysia | Hypertension | Hospitals (Public & Private) / Clinics (Public, Private & NGO\|Other) / Pharmacies (Public & Private) | Diagnosis / Chronic care management / Medicines | Narrative inquiry | 12 key informants, 24 health professionals involved in hypertension management and 37 patients with hypertension | Interviews |
| Ruiz-Sandoval et al. | 2018 | [Public and Private Hospital Care Disparities of Ischemic Stroke in Mexico: Results from the Primer Registro Mexicano de Isquemia Cerebral (PREMIER) Study](https://doi.org/10.1016/j.jstrokecerebrovasdis.2017.09.025) | J Stroke Cerebrovasc Dis | Mexico | Cardiovascular disease (stroke) | Hospitals (Public & Private) | Curative & Rehabilitative | Cohort (prospective) | 1023 adult patients in public or private hospital | Registry data, 2005/2006 |
| Rutebemberwa et al. | 2019 | [Pathways to diabetic care at hospitals in rural Eastern Uganda: a cross sectional study](http://dx.doi.org/10.1186/s12913-019-3873-z) | BMC Health Serv Res | Uganda | Diabetes | Clinics (Public & Private) / Pharmacies (Public & Private) | Diagnosis / Chronic care management / Medicines | Cross-sectional | 496 patients using care at public hospitals | Surveys, 2012/2013 |
| Saleh et al. | 2018 | [Health seeking for chronic lung disease in central Malawi: Adapting existing models using insights from a qualitative study](https://doi.org/10.1371/journal.pone.0208188) | PloS one | Malawi | Chronic respiritory disease | Clinics (Public & Private) / Hospitals (Public & Private) | Diagnosis / Chronic care management | Phenomenology | A. 14 patients using care at health facilities and 5 officers in charge/medical assistants and village chiefs, B. 4 community members, 2 informal health providers, 1 health surveillance assistants and 2 village health committees | A. Interviews and B. focus groups, 2015 |
| Sérvio et al. | 2019 | [Barriers to cardiac rehabilitation delivery in a low-resource setting from the perspective of healthcare administrators, rehabilitation providers, and cardiac patients](http://dx.doi.org/10.1186/s12913-019-4463-9) | BMC Health Serv Res | Brazil | Cardiovascular disease (acute myocardial infarction and stroke) | Clinics (Public & Private) / Hospitals (Public & Private) | Rehabilitative | Cross-sectional | 805 patients receiving cardiac rehabilitation and 32 healthcare administrators | Surveys, 2015-2017. |
| Shahtaheri et al. | 2022 | [Long-term cost-effectiveness of quality of diabetes care; experiences from private and public diabetes centers in Iran](http://dx.doi.org/10.1186/s13561-022-00377-9) | Health Econ Rev | Iran | Diabetes | Hospitals (Public & Private) | Diagnosis / Curative & Rehabilitative / Chronic care management / Medicines | Cohort (retrospective) | 1978 patients | Hospital data, unclear |
| Sharma et al. | 2016 | [Excess cost burden of diabetes in Southern India: a clinic-based, comparative cost-of-illness study](https://doi.org/10.1017/gheg.2016.2) | Glob Health Epidemiol Genom | India | Diabetes | Clinics (Public & Private) | Diagnosis / Chronic care management | Cross-sectional | 606 adult patients using care at public or private clinics and 356 non-patients | Surveys, 2010 |
| Shivashankar et al. | 2016 | [Adherence to diabetes care processes at general practices in the National Capital Region-Delhi, India](http://dx.doi.org/10.4103/2230-8210.180000) | Indian J Endocrinol Metab | India | Diabetes | Clinics (Public & Private) | Diagnosis / Chronic care management | Cross-sectional / Phenomenology | 406 patients from a specific sample of general practitioners, 23 physicians | Surveys (cross-verified with patient records if possible), 2011/2012 |
| Shrestha et al. | 2013 | [Cost of diabetes mellitus care among patients attending selected outpatient clinics](http://dx.doi.org/10.31729/jnma.2114) | J Nepal Med Assoc | Nepal | Diabetes | Clinics (Public & Private) | Chronic care management | Cross-sectional | 227 patient aged 20 to 60 years with >1 year of illness using care at outpatient clinics | Surveys, 2010 |
| Signorelli et al. | 2016 | [Socioeconomic disparities in access to a hepatocellular carcinoma screening program in Brazil](http://dx.doi.org/10.6061/clinics/2016(07)01) | Clinics | Brazil | Cancer (liver) | Hospitals (Public & Private) | Prevention & Screening | Cohort (retrospective) | 253 patients using care at public or private hospitals | Medical records, 2012-2014 to 10 years prior |
| Smythe et al. | 2022 | [Access to health care for people with stroke in South Africa: a qualitative study of community perspectives](http://dx.doi.org/10.1186/s12913-022-07903-9) | BMC Health Serv Res | South Africa | Cardiovascular disease (stroke) | Clinics (Public/Private) / Hospitals (Public/Private) | Diagnosis / Curative & Rehabilitative | Narrative inquiry | 16 persons with stroke | Interviews, 2020 |
| Soares et al. | 2020 | [Low overall survival in women with de novo metastatic breast cancer: Does this reflect tumor biology or a lack of access to health care?](http://dx.doi.org/10.1200/JGO.19.00408) | JCO Glob Oncol | Brazil | Cancer (breast) | Hospitals (Public & Private) | Curative & Rehabilitative | Cohort (retrospective) | 277 patients | Registry data, 1995-2011 |
| Subramanian et al. | 2018 | [Cost and affordability of non-communicable disease screening, diagnosis and treatment in Kenya: Patient payments in the private and public sectors](https://doi.org/10.1371/journal.pone.0190113) | PLoS One | Kenya | Multiple NCDs (4) - cancers (breast & cervical), cardiovascular disease, chronic respiratory diseases, diabetes | Clinics (Public & Private) / Hospitals (Public & Private) | Diagnosis / Curative & Rehabilitative / Chronic care management | Cross-sectional | Public hospital, private-sector practitioners (generally two clinicians for each disease area) and private hospitals | Hospital payment data, 2016 |
| Syed et al. | 2018 | [Access to Antihypertensive Medicines at the Household Level: A Study From 8 Counties of Kenya](http://dx.doi.org/10.1016/j.gheart.2018.08.001) | Glo Heart | Kenya | Hypertension | Clinics (Public & Private) / Pharmacies (Public & Private) | Medicines | Cross-sectional | 445 patients | Surveys & interviews, 2016 |
| Tejada-Tayabas et al. | 2015 | [Medical therapeutic itineraries of women with breast cancer diagnosis affiliated to the People's Health Insurance in San Luis PotosÃ­, central Mexico](https://doi.org/10.1590/0102-311X00009114) | Cad Saude Publica | Mexico | Cancer (breast) | Clinics (Public & Private) / Hospitals (Public & Private) | Diagnosis / Curative & Rehabilitative | Grounded theory | 12 patients affiliated to the People’s Health Insurance | Interviews, 2013 |
| Tekinturhan et al. | 2013 | [Improving access to care in low and middle-income countries: institutional factors related to enrollment and patient outcome in a cancer drug access program](http://dx.doi.org/10.1186/1472-6963-13-304) | BMC Health Serv Res | Multicountry (44) - Albania, Armenia, Azerbaijan, Barbados, Belarus, Benin, Bhutan, Burkina Faso, Cambodia, Cameroon, Côte d'Ivoire, Ethiopia, Fiji, Gabon, Georgia, Ghana, Haïti, Kazakhstan, Kenya, Kyrgyzstan, Madagascar, Mali, Mauritius, Moldova, Mongolia, Mozambique, Nepal, Niger, Nigeria, Republic of Congo, Rwanda, Saint ucia, Senegal, Seychelles, Sierra Leone, Sudan, Surinam, Tajikistan, Tanzania, Togo, Uganda, Uzbekistan, Zambia, Zimbabwe | Cancer (2) - leukemia and gastrointestinal | Hospitals (Public & Private) | Medicines | Cohort (retrospective) | 4946 patients who are not insured or reimbursed and cannot pay for treatment privately | Medical data, 2003-2010 |
| Thakur et al. | 2019 | [Determinants of Expenditure on Diabetes Care: A Community Based Longitudinal Study in aResettlement Colony of Delhi](https://pubmed.ncbi.nlm.nih.gov/31571452/) | J Assoc Physicians India | India | Diabetes | Clinics (Public & Private) / Pharmacies (Public & Private) | Diagnosis / Chronic care management / Medicines | Cohort (retrospective) | 150 patients aged 25+ using public hospital care | Interviews and patient diaries, 2014 |
| Thomson et al. | 2021 | [Applying an ecological framework to examine the multiple levels of influence affecting the utilisation of private sector adult asthma services in Khartoum, Sudan: a mixed methods study [version 1; peer review: 1 approved with reservations, 1 not approved]](http://dx.doi.org/10.12688/F1000RESEARCH.25417.1) | F1000 Res | Sudan | Chronic respiratory disease (Asthma) | Clinics (Private) / Hospitals (Private) / Phamarcies (Private) | Diagnosis / Chronic care management / Medicines | Cross-sectional / Narrative inquiry | A. 74 private facilities (Hospitals, clinics, pharmacies) and B. 14 adult patients seeking care in private facilities | A. Surveys and B. interviews, 2014/2015 |
| Tolla et al. | 2017 | [Out-of-pocket expenditures for prevention and treatment of cardiovascular disease in general and specialised cardiac hospitals in Addis Ababa, Ethiopia: a cross-sectional cohort study](http://dx.doi.org/10.1136/bmjgh-2016-000280) | BMJ glob. health | Ethiopia | Cardiovascular disease | Hospitals (Public & Private) | Diagnosis / Curative & Rehabilitative / Chronic care management | Cross-sectional | 589 adult patients using hospital care (newly diagnosed excluded) | Surveys, 2015 |
| Tripathy et al. | 2016 | [Cost of hospitalisation for non-communicable diseases in India: are we pro-poor?](http://dx.doi.org/10.1111/tmi.12732) | Trop Med Int Health | India | Multiple NCDs (4) - cancers, cardiovascular disease, chronic respiratory disease, endocrine disorders, psychiatric and neurological disorders | Hospitals (Public & Private) | Diagnosis / Curative & Rehabilitative / Chronic care management | Cross-sectional | 11,843 adult hospitalized patients | Survey data from National Sample Survey Office, 2014 |
| Tripathy et al. | 2018 | [Cost of diabetic care in India: An inequitable picture](https://doi.org/10.1016/j.dsx.2017.11.007) | Diabetes Metab Syndr | India | Diabetes | Clinics (Private) / Hospitals (Private) / Phamarcies (Private) | Diagnosis / Curative & Rehabilitative / Chronic care management | Cross-sectional | 915 hospitalized adult patients and 3935 patients receiving outpatient care | Survey data from National Sample Survey Office, 2014 |
| Tumanan-Mendoza et al. | 2018 | [Economic burden of hospitalisation for congestive heart failure among adults in the Philippines](http://dx.doi.org/10.1136/heartasia-2018-011039) | Heart Asia | Philippines | Cardiovascular disease | Hospitals (Public & Private) / Pharmacies (Public & Private) | Diagnosis / Curative & Rehabilitative | Cross-sectional | 34 private and public hospitals | Hospital data, 2014 |
| Tusubira et al. | 2020 | [Accessing medicines for non-communicable diseases: Patients and health care workers' experiences at public and private health facilities in Uganda](http://dx.doi.org/10.1371/journal.pone.0235696) | PLoS ONE | Uganda | Multiple NCDs (2) - diabetes and hypertension | Clinics (Public & Private) | Medicines | Narrative inquiry | 36 and 128 patients using outpatient care and 26 healthcare workers and patient association leaders | A. Interviews, B. focus group discussions, 2016/2017 |
| Yadav et al. | 2021 | [Socioeconomic Impact of Hospitalization Expenditure for Treatment of Noncommunicable Diseases in India: A Repeated Cross-Sectional Analysis of National Sample Survey Data, 2004 to 2018](https://doi.org/10.1016/j.vhri.2020.12.010) | Value Health Reg Issues | India | Multiple NCDs (4) - cancers, cardiovascular disease & stroke, chronic respiratory disease, diabetes | Hospitals (Public & Private) | Diagnosis / Curative & Rehabilitative | Cross-sectional | 383,338, 333,104 & 555,115 individuals from general population | Survey data from national sample surveys (NSS), 2004, 2014, 2018 |

Table S. 2 – Research designs and Mixed Methods Appraisal Tool (MMAT) ratings of selected studies

|  | Number of studies | Sample size | | | | MMAT rating | | | | |
| --- | --- | --- | --- | --- | --- | --- | --- | --- | --- | --- |
| Design Type |  | ≤500 | >500  ≤1,500 | >1,500  ≤10,000 | >10,000 | ≤50% | >50%  ≤70% | >70%  ≤90% | >90% | Avg. |
| Cross sectional | 62 | 18 | 22 | 13 | 9 | 4 | 12 | 30 | 16 | 83% |
| Cohort | 17 | 10 | 3 | 3 | 1 | 1 | 3 | 8 | 5 | 86% |
| Quasi experimental | 2 | 0 | 0 | 0 | 2 | 0 | 0 | 0 | 2 | 100% |
| Random control trial | 1 | 1 | 0 | 0 | 0 | 0 | 0 | 0 | 1 | 100% |
| Mixed methods | 14 | 11 | 2 | 0 | 1 | 1 | 3 | 6 | 4 | 83% |
| Qualitative | 19 | 19 | 0 | 0 | 0 | 2 | 3 | 8 | 6 | 84% |
|  | 115 | 59 | 27 | 16 | 13 | 8 | 21 | 52 | 34 |  |

Table S. 3 – PRISMA Checklist

| **Section and Topic** | **Item #** | **Checklist item** | **Location where item is reported** |
| --- | --- | --- | --- |
| **TITLE** | | |  |
| Title | 1 | Identify the report as a systematic review. | p.1 |
| **ABSTRACT** | | |  |
| Abstract | 2 | See the PRISMA 2020 for Abstracts checklist. | p.2 |
| **INTRODUCTION** | | |  |
| Rationale | 3 | Describe the rationale for the review in the context of existing knowledge. | p.3 |
| Objectives | 4 | Provide an explicit statement of the objective(s) or question(s) the review addresses. | p.4 |
| **METHODS** | | |  |
| Eligibility criteria | 5 | Specify the inclusion and exclusion criteria for the review and how studies were grouped for the syntheses. | p.5 |
| Information sources | 6 | Specify all databases, registers, websites, organisations, reference lists and other sources searched or consulted to identify studies. Specify the date when each source was last searched or consulted. | p.6 and Supplementary Material Text S.1 |
| Search strategy | 7 | Present the full search strategies for all databases, registers and websites, including any filters and limits used. | p.6 |
| Selection process | 8 | Specify the methods used to decide whether a study met the inclusion criteria of the review, including how many reviewers screened each record and each report retrieved, whether they worked independently, and if applicable, details of automation tools used in the process. | p.6 |
| Data collection process | 9 | Specify the methods used to collect data from reports, including how many reviewers collected data from each report, whether they worked independently, any processes for obtaining or confirming data from study investigators, and if applicable, details of automation tools used in the process. | pp.5-7 |
| Data items | 10a | List and define all outcomes for which data were sought. Specify whether all results that were compatible with each outcome domain in each study were sought (e.g. for all measures, time points, analyses), and if not, the methods used to decide which results to collect. | pp.5-7 |
|  | 10b | List and define all other variables for which data were sought (e.g. participant and intervention characteristics, funding sources). Describe any assumptions made about any missing or unclear information. | pp.5-7 |
| Study risk of bias assessment | 11 | Specify the methods used to assess risk of bias in the included studies, including details of the tool(s) used, how many reviewers assessed each study and whether they worked independently, and if applicable, details of automation tools used in the process. | p.6 |
| Effect measures | 12 | Specify for each outcome the effect measure(s) (e.g. risk ratio, mean difference) used in the synthesis or presentation of results. | pp.5-7 |
| Synthesis methods | 13a | Describe the processes used to decide which studies were eligible for each synthesis (e.g. tabulating the study intervention characteristics and comparing against the planned groups for each synthesis (item #5)). | pp.5-7 |
|  | 13b | Describe any methods required to prepare the data for presentation or synthesis, such as handling of missing summary statistics, or data conversions. | pp.5-7 |
|  | 13c | Describe any methods used to tabulate or visually display results of individual studies and syntheses. | pp.5-7 |
|  | 13d | Describe any methods used to synthesize results and provide a rationale for the choice(s). If meta-analysis was performed, describe the model(s), method(s) to identify the presence and extent of statistical heterogeneity, and software package(s) used. | pp.5-7 |
|  | 13e | Describe any methods used to explore possible causes of heterogeneity among study results (e.g. subgroup analysis, meta-regression). | pp.5-7 |
|  | 13f | Describe any sensitivity analyses conducted to assess robustness of the synthesized results. | pp.5-7 |
| Reporting bias assessment | 14 | Describe any methods used to assess risk of bias due to missing results in a synthesis (arising from reporting biases). | pp.5-7 |
| Certainty assessment | 15 | Describe any methods used to assess certainty (or confidence) in the body of evidence for an outcome. | pp.5-7 |
| **RESULTS** | | |  |
| Study selection | 16a | Describe the results of the search and selection process, from the number of records identified in the search to the number of studies included in the review, ideally using a flow diagram. | pp.7-8 |
|  | 16b | Cite studies that might appear to meet the inclusion criteria, but which were excluded, and explain why they were excluded. | pp.7-8 |
| Study characteristics | 17 | Cite each included study and present its characteristics. | Supplementary Material Table S.1 |
| Risk of bias in studies | 18 | Present assessments of risk of bias for each included study. | p.11 and Supplementary Material Table S.2 |
| Results of individual studies | 19 | For all outcomes, present, for each study: (a) summary statistics for each group (where appropriate) and (b) an effect estimate and its precision (e.g. confidence/credible interval), ideally using structured tables or plots. | pp.7-14 |
| Results of syntheses | 20a | For each synthesis, briefly summarise the characteristics and risk of bias among contributing studies. | pp.7-14 |
|  | 20b | Present results of all statistical syntheses conducted. If meta-analysis was done, present for each the summary estimate and its precision (e.g. confidence/credible interval) and measures of statistical heterogeneity. If comparing groups, describe the direction of the effect. | pp.7-14 |
|  | 20c | Present results of all investigations of possible causes of heterogeneity among study results. | pp.7-14 |
|  | 20d | Present results of all sensitivity analyses conducted to assess the robustness of the synthesized results. | pp.7-14 |
| Reporting biases | 21 | Present assessments of risk of bias due to missing results (arising from reporting biases) for each synthesis assessed. | pp.7-14 |
| Certainty of evidence | 22 | Present assessments of certainty (or confidence) in the body of evidence for each outcome assessed. | pp.7-14 |
| **DISCUSSION** | | |  |
| Discussion | 23a | Provide a general interpretation of the results in the context of other evidence. | pp.14-15 |
|  | 23b | Discuss any limitations of the evidence included in the review. | pp.16-17 |
|  | 23c | Discuss any limitations of the review processes used. | p.17 |
|  | 23d | Discuss implications of the results for practice, policy, and future research. | p.15-16 |
| **OTHER INFORMATION** | | |  |
| Registration and protocol | 24a | Provide registration information for the review, including register name and registration number, or state that the review was not registered. | p.4 |
|  | 24b | Indicate where the review protocol can be accessed, or state that a protocol was not prepared. | p.4 |
|  | 24c | Describe and explain any amendments to information provided at registration or in the protocol. | p.4 |
| Support | 25 | Describe sources of financial or non-financial support for the review, and the role of the funders or sponsors in the review. | p.19 |
| Competing interests | 26 | Declare any competing interests of review authors. | p.19 |
| Availability of data, code and other materials | 27 | Report which of the following are publicly available and where they can be found: template data collection forms; data extracted from included studies; data used for all analyses; analytic code; any other materials used in the review. | p.19 |

Source: Page MJ, McKenzie JE, Bossuyt PM, Boutron I, Hoffmann TC, Mulrow CD, et al. The PRISMA 2020 statement: an updated guideline for reporting systematic reviews. BMJ 2021;372:n71. doi: 10.1136/bmj.n71
